# Regularized Joint Maximum Likelihood Estimation of Latent Space Item Response Models

**DOI:** 10.1017/psy.2025.10068

**Published:** 2026-01-09

**Authors:** Dylan Molenaar, Minjeong Jeon

**Affiliations:** 1 Department of Psychology, University of Amsterdam, The Netherlands; 2 Department of Education, University of California, Los Angeles, USA

**Keywords:** cross-validation, item response theory, latent space modeling, maximum likelihood

## Abstract

In latent space item response models (LSIRMs), subjects and items are embedded in a low-dimensional Euclidean latent space. As such, interactions among persons and/or items can be revealed that are unmodeled in conventional item response theory models. Current estimation approach for LSIRMs is a fully Bayesian procedure with Markov Chain Monte Carlo, which is, while practical, computationally challenging, hampering applied researchers to use the models in a wide range of settings. Therefore, we propose an LSIRM based on two variants of regularized joint maximum likelihood (JML) estimation: penalized JML and constrained JML. Owing to the absence of integrals in the likelihood, the JML methods allow for various models to be fit in limited amount of time. This computational speed facilitates a practical extension of LSIRMs to ordinal data, and the possibility to select the dimensionality of the latent space using cross-validation. In this study, we derive the two JML approaches and address different issues that arise when using maximum likelihood to estimate the LSIRM. We present a simulation study demonstrating acceptable parameter recovery and adequate performance of the cross-validation procedure. In addition, we estimate different binary and ordinal LSIRMs on real datasets pertaining to deductive reasoning and personality. All methods are implemented in R package ‘LSMjml’ which is available from CRAN.

The latent space item response model (LSIRM) integrates latent space models from social network analysis (Hoff et al., 2002) with item response theory (IRT) from psychometrics, extending traditional IRT by embedding persons and items in a metric, multidimensional latent space. As such, LSIRMs may reveal item–person interactions that generally remain unnoticed in conventional models giving more insights about residual dependencies between persons, between items, and between items and persons. This has been shown valuable in the substantive fields of intelligence (Kang & Jeon, 2024; Kim et al., 2014), developmental psychology (Go et al., 2022), mental health (Jeon & Schweinberger, 2024), social influence (Park et al., 2023), national school policy (Jin et al., 2022), and student monitoring (Jeon et al., 2021). In addition, extensions of the LSIRM have enabled the analysis of multilevel structured data (Jin et al., 2022), longitudinal data (Jeon & Schweinberger, 2024; Park et al., 2022), and response time data (Jin et al. 2023; Kang & Jeon, 2024).

Estimation of LSIRMs has been dominated by the fully Bayesian, Markov Chain Monte Carlo (MCMC) estimation scheme by Jeon et al. (2021), which has been implemented in R (Go et al., 2023), JAGS, Stan, NIMBLE (Luo et al., 2023), and Shiny (Ho & Jeon, 2023). Although valuable due to its flexibility and its facilities for posterior diagnostics, the MCMC routines are numerically demanding, which may hamper (applied) researchers from using the LSIRM in a wide range of settings that may involve large-scale data. In addition, due to the numerical demanding nature of the MCMC approach, model fit comparison is relatively challenging as it involves multiple models to be fit to the data. Although there are alternatives like leave-one-out cross-validation using Pareto smoothing (Vehtari et al., 2017), these will still be computationally intensive for models like the LSIRM. Currently, researchers rely on spike and slab priors for model selection (e.g., George & McCulloch, 1997; Ishwaran & Rao, 2005) to compare a given LSIRM with 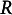 dimensions to a baseline model with 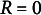 . This approach is feasible in the MCMC framework as it does not increase the computational burden significantly. However, tools to compare multiple competing models that differ in 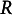 have not yet been developed.

Therefore, in this study, we propose different joint maximum likelihood (JML)-based approaches to fit various LSIRMs to data in a fast and efficient way, facilitating large-scale model application and model selection. In the early years of IRT, JML (Birnbaum, 1968; Lord, 1980; Mislevy & Stocking, 1989) was one of the dominant approaches to fit conventional IRT models to data using software packages such as LOGIST (Wingersky, 1983) and BICAL (Wright & Mead, 1976). As computers were not as fast as nowadays, a desirably practical property of JML was its numerical efficiency. That is, in JML, all parameters are assumed to be fixed effects so that the likelihood function does not include any integrals. These integrals make approaches such as MCMC and marginal maximum likelihood (MML; Bock & Aitkin, 1981) relatively time-consuming, as they require numerical approximation due to the lack of a closed form solution. However, over the years, popularity of JML decreased in favor of MML up until recently, when JML was revived in IRT by the work of Chen et al. (2019, 2020) and Bergner et al. (2022). In this work, the authors developed variations of JML that are suitable for estimation of high-dimensional IRT models on large datasets, which is—even with today’s computers—still challenging for the state-of-the art MCMC and MML approaches. In addition to IRT, some JML approaches have been developed for latent space models for social network analysis. That is, Zhang et al. (2022) and Ma et al. (2020) focused on a latent space model with high-dimensional covariates and JML estimation of its parameters. In the approach by Zhang et al., the covariates enter the model via a generalized linear latent variable model, whereas in Ma et al., these covariates are included as predictors next to the latent space positions.

As the IRT models estimated by JML are typically high-dimensional, the complexity of these models is commonly managed through regularization. Regularization is a technique originating from ridge regression (Hoerl & Kennard, 1970) in which the regression parameters are pushed to zero to prevent overfitting and to stabilize parameter estimates in the case of multicollinearity. Currently, regularization includes a variety of techniques, such as penalization and constraints on the parameter space, to promote parameter shrinkage (Hastie et al., 2009; Tibshirani, 1996) or to improve finite-sample performance (Firth, 1993). For example, Chen et al. (2019) used constraints on the parameter space to estimate a multidimensional exploratory IRT model, and Bergner et al. (2022) used 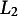 penalization of the parameter space of a general family of IRT models for collaborative filtering.

In this study, we apply these two regularization strategies, constraining the parameter space and penalizing regions of the parameter space, to provide an efficient and stable JML estimation algorithm for the relatively complex LSIRM model. As the effects of regularization are comparable to the effects of parameter priors on the likelihood function, we will show that our JML-based LSIRM is a special cases of the existing MCMC-based LSIRM, providing a highly comparable but easier to estimate variant of the LSIRM. As MCMC obviously has other practical advantages over JML (e.g., flexibility and full posterior information), we present our JML approach as an extension of the current LSIRM modeling toolbox, not as an alternative.

The models by Zhang et al. (2022) and Ma et al. (2020) discussed above are related but different from our approach. That is, both Zhang et al. and Ma et al. focused on a unipartite (only modeling one set of nodes, e.g., persons but not items) latent space model without IRT component, and an inner product distance measure. Our model, however, is bipartite, includes an IRT model component, and uses the Euclidean distance. All three of these aspects make our study to face very different challenges in the development of the JML approach. However, besides differences in the underlying model, our estimation approach and that of Zhang et al. and Ma et al. are similar in spirit.

One of the key issues that arises in implementing a maximum likelihood scheme for the LSIRM is that the model can only be identified up to a rotation of the latent space. In the MCMC framework by Jeon et al. (2021), researchers addressed this problem by post-processing the MCMC chains using Procrustes matching (Gower 1975; Sibson, 1979), which involves rotating the latent space to the space from the MCMC iteration with the highest likelihood. Such an approach is infeasible in a maximum likelihood framework. We therefore propose alternative constraints based on the echelon structure from exploratory factor analysis (Dolan et al., 2009; McDonald, 2013). As these constraints are specified a priori, this approach has the advantage that the orientation on which the results are produced is explicitly defined, which facilitates comparisons to other methods.

Thus, using JML, the LSIRM can be estimated in a computationally fast and efficient way, allowing researchers to fit different LSIRM models in a limited amount of time. A resulting advantage that we demonstrate in this study is that the selection of the dimensionality of the latent space can be informed by a 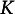 -fold cross-validation routine (e.g., Bergner et al., 2022; Haslbeck & van Bork, 2024). As discussed above, it is currently not possible to compare models differing in the dimensions of the latent space. This possibility, therefore, seems a valuable addition to the toolbox of researchers interesting in LSIRM modeling. In addition, we demonstrate how it is straightforward to use our approach to fit LSIRMs to ordinal data in a limited amount of time. Ordinal LSIRM have recently been development in an MCMC framework (see De Carolis et al., 2025) but are time-consuming to fit.

The outline of this article is as follows: We first present the LSIRM and discuss MCMC estimation of the model parameters. Next, we present the two JML variants and discuss the constraints needed to solve the rotational indeterminacy of the latent space. Then, we outline the methods for parameter estimation, a generalization to ordinal data, and a cross-validation approach to model selection. In the simulation study, we demonstrate that the accuracy of the parameter recovery of our JML approach is comparable to that of the MCMC approach and that the cross-validation approach successfully selects the correct model in most cases. Next, in two illustrations, we apply a binary LSIRM to a dataset on deductive reasoning and an ordinal LSIRM to a dataset on personality. We end with a general discussion.

## 1 Latent space item response models

The LSIRM is a statistical model for the dichotomous item responses 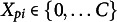 of person 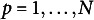 on item 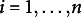 . It is assumed that, after accounting for the main effect of the person by person intercept 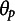 , and for the main effect of the item by item intercept 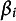 , the person and item residuals can be embedded in an 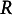 -dimensional Euclidean latent space using the 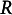 -dimensional vector of person coordinates 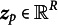 and the 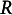 -dimensional vector of item coordinates 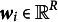 . As a result, the conditional probability, 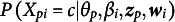 , is given by
(1)

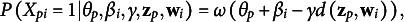

 where 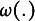 is a logistic function, 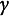 is the strictly positive weight parameter, and 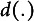 is a distance function. Even though 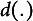 can be any distance function that obeys to the mathematical principles of reflexivity, symmetry, and triangular inequality (e.g., Chebyshev distance, Minkowski distance, and Manhattan distance), LSIRMs have generally been applied using the Euclidian distance, that is,
(2)

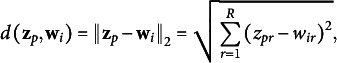

 where 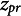 and 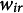 are the 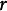 th element of 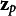 and 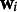 , respectively. In the present framework, 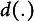 is a distance function; however, it can be any other function that models the relation of the latent space coordinates. For instance, Zhang et al. (2022) and Ma et al. (2020) used an inner product similarity measure for 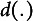 , which would give 
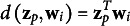
 . The advantage of the inner product is that it is of low rank, resulting in tractable theoretical properties of the statistical model. However, the inner product cannot be interpreted as a distance that is undesirable for the present aim. See Jeon et al. (2021) for a discussion of the interpretational challenges of the inner product measure in LSIRMs.

The LSIRM above can be interpreted as a Rasch model (Rasch, 1960) in which the residuals are further modeled using 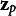 and 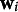 . As a result, the LSIRM can give more insight in local dependencies of the item response data due to interactions between items, interactions between persons, or interactions between items and persons. For instance, such interactions may arise because of some items requiring a more similar response processes than others, some persons sharing a relevant background characteristic more than others (e.g., educational attainment and motivation), and some persons use a different solution strategy on some of the items than others.

### 1.1 Estimation

MCMC sampling methods have been developed to fit the LSIRM (see, e.g., Jeon et al., 2021). By specifying prior distributions for the model parameters presented above, it is possible to estimate an additional nonnegative parameter 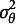 , the variance of the person intercept. In addition, 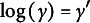 is modeled to ensure that 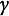 is strictly positive. Next, if we collect all 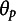 parameters in the 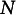 -dimensional vector 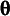 and all 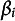 parameters in in the 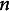 -dimensional vector 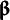 , and if we denote the full 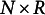 matrix of stacked 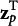 vectors by 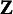 , and the full 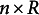 matrix of stacked 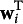 vectors by 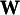 , the posterior distribution of the model parameters is given by
(3)

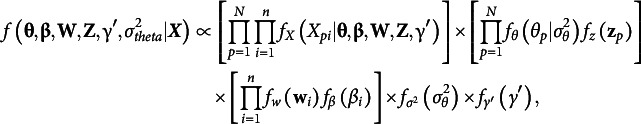

 where 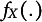 is the Bernoulli distribution with success probability given by Equation (1), and where 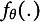 , 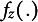 , 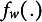 , 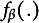 , 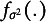 , and 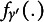 are (hyper) prior distributions for, respectively, 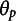 , 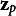 , 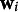 , 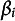 , 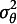 , and 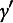 . In general, the following distributions are used (e.g., Go et al., 2023; Jeon et al., 2021): a univariate normal for 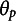 with mean 0 and variance 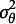 (as discussed above), a univariate normal for 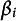 with mean 0 and variance 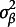 , a multivariate orthogonal standard normal distributions for 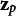 and 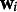 , an inverse-gamma distribution for 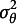 with shape parameter 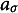 and scale parameter 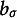 , and a normal distribution for 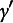 with mean 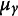 and variance 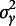 .

### 1.2 Identification

The item and person intercept parameters 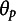 and 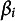 are readily identified by fixing the prior mean of 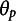 to 0 in the above. For 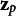 and 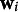 , fixing the 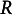 dimensions to be orthogonal with zero mean and unit variance ensures that the locations of the 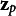 and 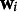 coordinates are uniquely identified. However, there still exists a rotational indeterminacy. That is, any two of the 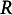 dimensions of 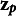 and 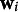 can be rotated by an arbitrary angle 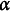 , resulting in a different solution with the same Euclidean distances 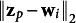 and the same data likelihood. In the MCMC framework to fit the LSIRM, this indeterminacy causes each sample from the posterior parameter distribution to be potentially subject to a different rotation. Posterior sample means are therefore confounded and cannot be used as parameter estimates. To solve this issue, researchers rely on Procrustes matching (Gower, 1975; Sibson, 1979) in which each posterior sample of 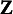 and 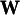 is transformed to match a given target, respectively, 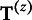 and 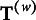 . In general, these target matrices are chosen to be the 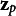 and 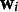 samples from the MCMC iteration with the largest likelihood (Jeon et al., 2021).

### 1.3 Model selection

For model selection, Jeon et al. (2021) proposed the use of a spike and slab prior for 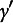 . Specifically, instead of a normal prior, 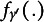 is specified as a mixture of two normal distributions

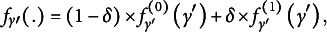

 where 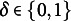 is a dichotomous parameter, 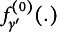 is the spike prior with mean 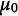 and variance 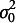 , and 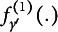 is the slab prior with mean 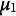 and variance 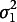 . The spike prior mean and variance should be chosen so that the resulting distribution of untransformed 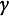 has a mean close to 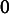 and a small variance, while the slab prior mean and variance should reflect the more conventional prior mean and variance. Next, by assuming 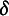 to be Bernoulli distributed with success parameter 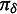 , and by specifying a Beta prior for 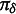 , parameter 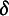 can be sampled along with the other parameters in the model. Model selection then involves the posterior probability that 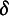 is equal to 1. If this probability is large (commonly a cutoff of 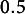 is used), it is concluded that the LSIRM with the 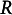 under consideration accounts better for the data than an IRT model without latent space (i.e., a one-parameter logistic model).

## 2 Joint maximum likelihood estimation

As discussed, the MCMC estimation scheme above can be time-consuming as sufficient samples need to be drawn from the posterior parameter distribution. To have a fast alternative available, below we present a JML estimation approach to estimate the parameters from the LSIRM. Implementing the LSIRM in a maximum likelihood framework brings specific identification challenges, which we address below. In addition, we discuss a model selection procedure based on 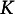 -fold cross-validation that is impractical or infeasible for the more time-consuming estimation algorithms, and we present a generalization to ordinal data.

In maximum likelihood estimation, the parameters of a statistical model are estimated by maximizing the likelihood of the data for the unknown model parameters. For LSIRM, this would involve the joint log-likelihood function, 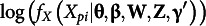 with 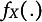 from Equation (3), summed over items and persons. However, without further constraints, this model is unidentified. First of all, in a JML framework, all parameters are fixed effects so that 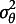 is not a model parameter as it is absorbed in the 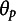 estimates. For the same reason, 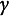 is not estimable as it is equivalent to the standard deviation of 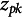 and 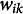 . Thus, 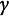 is absorbed in the 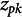 and 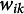 estimates. Therefore, the JML-based LSIRM is given by
(4)

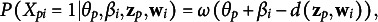

 where 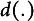 is the Euclidean distance from Equation (2).

As discussed above, to introduce further sparsity into the model, we consider two different regularization approaches based on either penalizing the joint likelihood or on constraining the maximum norm of the parameter vectors in the joint likelihood. As both regularization effects can be conceived as the effects of prior distributions, we intend to enhance the comparability of the JML results and the MCMC results. Both JML variants are discussed below.

### 2.1 Penalized joint maximum likelihood

In the first approach referred to as penalized joint maximum likelihood (pJML), we use an 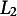 regularization penalty in the likelihood function. Although there are other options possible, we use the 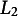 due to its correspondence to a normal prior. The log-likelihood function we consider is
(5)

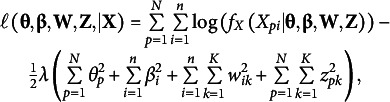

 where 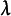 is a penalty parameter and 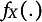 is a Bernoulli distribution with success probability given by Equation (4). Although, in the context of predictive accuracy, 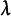 can be optimized using cross-validation, here we take 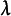 to be a prespecified parameter reflecting the precision of a zero-centered normal prior distribution. In the remainder of this article, we use 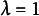 so that the regularization effect on 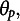 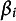 , 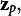 and 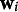 is comparable to that of standard normal priors typically used in MCMC estimation of the LSIRM. For instance, in the R package *lsirm12pl* (Go et al., 2023), which we use to compare our results to in the simulation study below, standard normal priors are used by default for 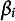 , 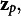 and 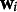 ( 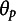 has a normal prior with variance 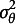 as discussed above).

The resulting approach can be seen as a variant of the Bayesian joint modal estimation approach used by Swaminathan and Gifford (1982, 1985, 1986) to fit one-, two-, and three-parameter logistic IRT models in a Bayesian framework. That is, Equation (5) is proportional to the log-posterior distribution of the parameters by which its maximum gives joint modal estimates of the parameters (i.e., MAP estimates). Moreover, the pJML approach in Equation (5) is a special case of the MCMC-based LSIRM in Equation (3). That is, fixing 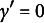 , 
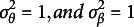
 in the logarithm of Equation (3), the MCMC-based LSIRM is equivalent to the pJML model in Equation (5) with 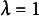 . The key difference that arises in practice is that MCMC focusses on posterior means instead of posterior modes by which the pJML model can be estimated much faster.

### 2.2 Constrained joint maximum likelihood

A next option referred to as constrained joint maximum likelihood (cJML) is to regularize the LSIRM model in Equation (4) by constraining the maximum norm of the person and item parameter vectors to some prespecified value. This approach has been proposed by Chen et al. (2019) for JML estimation of the multidimensional two-parameter logistic IRT model. Thus, we constrain the vector of person parameters 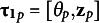 and the vector of item parameters 
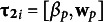
 in the following way:
(6)

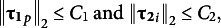

 where 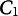 and 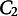 are the prespecified maximum parameter norms for the person and item parameter vectors, respectively, and 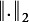 is the Euclidean norm. Thus, if 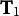 denotes a matrix of stacked 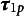 vectors and 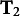 denotes a matrix of stacked 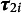 vectors, the likelihood function in this approach is given by
(7)

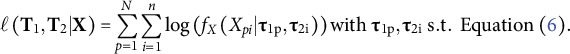



For a 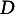 -dimensional two-parameter logistic IRT model, Chen et al. (2019) proposed 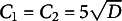 . As both 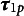 and 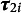 have 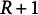 dimensions in our LSIRM model, we replace 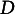 by 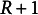 . In addition, we use 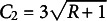 for the item parameters and 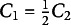 for the person parameters. Below, we show that these constraints are still less restrictive compared with the MCMC and pJML approaches.

The cJML model in Equation (7) can similarly be conceived as a joint modal estimation approach. To this end, the constraints in Equation (6) are enforced by using priors 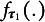 and 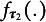 on 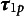 and 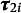 , respectively. These priors are uniform on an ( 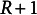 )-dimensional Euclidean ball with radius 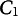 for the person parameters and 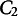 for the item parameters, that is,
(8)

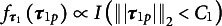

 and
(9)

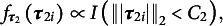

 where 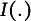 is the indicator function. Although, in this study, we rely on Equation (7), the above joint modal formulation of the cJML model is equivalent and explicates the difference with the pJML and MCMC approaches, both of which use normal priors. Unlike the pJML, the cJML-based LSIRM model is not strictly a special case of the MCMC-based LSIRM with standard normal priors discussed above. However, it is a special case in the more general framework of Equation (3) with 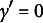 and 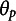 and 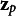 following the prior in Equation (8) and 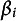 and 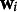 following the prior in Equation (9).

To see that the cJML approach is the least restrictive approach in terms of parameter regularization, we compare the prior variance across the three approaches. For the MCMC and pJML approaches, all priors have variance 1 (except for 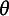 in the MCMC approach). The variances of the priors in Equations (8) and (9) are 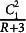 for the person parameters and 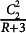 for the item parameters. Given our choices for 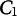 and 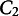 above, this results in a prior variance of 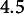 , 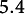 , and 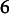 for the item parameters and of 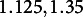 , and 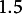 for the person parameters for 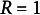 , 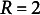 , and 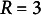 , respectively. These priors are, thus, less informative in terms of prior variance than those of the MCMC and pJML approaches (although differences are small for the person parameters). This will also be illustrated in the simulation study.

### 2.3 Consistency

Traditional unregularized JML is known to be theoretically inconsistent (Andersen, 1973; Haberman, 1977) as asymptotic theory is violated because of the number of parameters increasing with 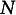 . In this study, it is expected that, due to the known correspondence of our 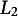 penalization to the effects of normal parameter priors, the finite-sample properties of the JML procedure will improve. However, we are unaware of theoretical proofs of consistency of pJML in the literature, so whether penalized JML is strictly consistent in the asymptotic sense has yet to be established.

For the cJML approach described above, it is known that the procedure is theoretically consistent under a double-asymptotic regime for structured generalized latent factor models (Chen et al., 2020) and multidimensional unstructured two-parameter IRT models (see Chen et al., 2019; Chen & Li, 2024). Similarly, approaches related to cJML are shown to be consistent for unipartite latent space models with low-rank inner-product distances (Ma et al., 2020; Zhang et al., 2022). However, these results do not necessarily apply to the present model due to the unstructured nature of the latent space, the nonlinear and high-rank character of the natural parameter arising from Euclidean distances, and the bipartite nature of our item–respondent interactions. As the JML consistency issues are known to decrease with an increasing number of items (Haberman, 1977), and our simulation study below shows that our JML approaches perform comparable to the existing Bayesian approaches, we believe that any inconsistency (if any) is relatively unproblematic given the aim of the present study.

### 2.4 Rotation

As discussed above, the distances in Equation (2) are subject to a rotational indeterminacy and can be arbitrarily rotated to produce the same distances. For instance, for 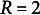 ,

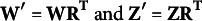

 with

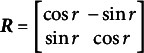

 will result in the same Euclidean distances 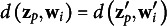 for all 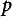 and all 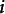 for any arbitrary angle of rotation 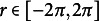 . As discussed above, this problem is solved in an MCMC framework by Procrustes matching in which the samples from the posterior are all transformed to a target solution. As our approaches do not involve posterior samples, we will use specific constraints on 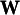 to fix 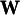 and 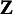 to an arbitrary rotation without affecting the data likelihood.

Specifically, the problem of rotational indeterminacy of **W** and 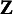 is similar to the problem of rotational indeterminacy of the factor loadings and the factor scores in factor analysis (e.g., Jennrich, 1978). Therefore, we will rely on what is referred to as “echelon rotation” in the factor analysis literature (Dolan et al., 2009; McDonald, 2013). That is, we will fix the 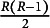 elements of the upper triangle of submatrix 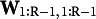 to 0. For instance, for 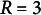 ,
(10)

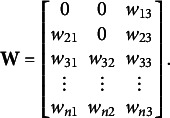



An advantage of using this constraint compared with Procrustes matching is that the solution is more explicitly defined and comparable across different methods that fit the LSIRM. As for Procrustes matching, the solution depends on the largest likelihood encountered during MCMC sampling, the solution may depend on the sampling scheme, estimation algorithm, or even on random fluctuations within the same method applied to the same dataset. This is unproblematic for a single application as any rotation solution is arbitrary. However, if applications or algorithms need to be compared, using Procrustes matching may confound the comparison. Using echelon rotation, any solution obtained with any estimation algorithm (MCMC or maximum likelihood, but also potential other procedures such as least squares, variational inference, and minorization) can be rotated to the structure above to facilitate comparison.

For 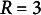 , the following three rotations are carried out to transform 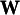 to an echelon structure:
(11)

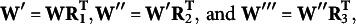

 where 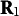 , 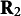 , and 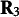 are given by
(12)

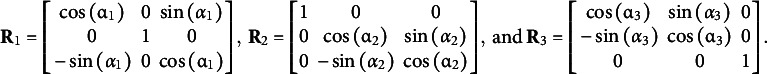



**Figure 1:**
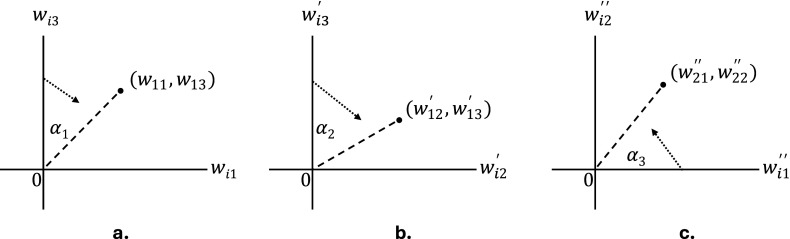
Graphical illustration of the echelon rotation for 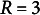 . The dotted arrows indicate the direction of the rotation, the solid dot denotes a specific coordinate 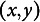 , and the striped lines give an indication of the new (i.e., rotated) position of the axis connected to the dotted arrow. The angle of rotation is indicated by 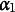 , 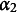 , and 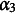 .

The angles of rotation 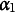 , 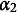 , and 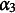 can be obtained by standard trigonometry and are given by
(13)

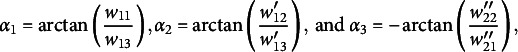

 where 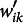 and 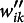 are the elements from the rotated 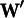 and 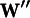 matrices. See Figure 1 for a graphical illustration of the rotation scheme.

That is, in the figure, it can be seen that, first, 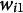 and 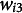 are rotated around 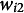 using angle 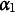 so that 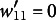 . Next, 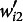 and 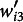 are rotated around 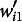 using angle 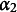 so that 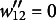 . Finally, 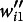 and 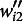 are rotated around 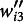 using angle 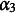 so that 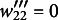 . Final matrix 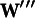 will have the structure in Equation (10). If 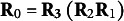 is denoted to be the overall rotation matrix, then the same rotation can be applied to 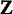 , resulting in 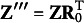 . As noted before, the Euclidean distances of the original parameters from 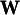 and 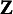 are equivalent to the Euclidean distances of the rotated parameters from 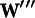 and 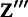 , that is, 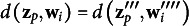 for all 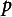 and all 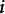 . Generalization to any 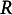 is straightforward and can be practically performed using the algorithm by, for instance, Wansbeek and Meijer (2000).

### 2.5 Model selection

Owing to its diagnostic character, in many situations, an LSIRM with 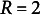 will suffice because of its suitability to visualize the results in 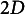 plots. However, if a more statistical informed decision needs to be made about 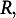 there are no methods available yet. In a maximum likelihood framework, direct comparison of the models using common fit indices, such as Akaike information criterion and Bayesian information criterion, is challenging because of the ambiguity about the definition of the parameter penalty for models with different dimensions of 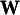 and 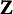 . As we rely on JML estimation, a suitable fit index may be provided by the Joint-likelihood-based information criteria (JIC) by Chen and Li (2022). However, in simulations, it turned out that the JIC only works for our model if the ratio 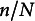 is larger than considered here (see below and Appendix B of the Supplementary Material).

Therefore, we propose a model selection procedure based on 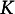 -fold cross-validation. Similar procedures have been proposed for model selection among multidimensional two-parameter logistic models (Bergner et al., 2022) and network models, including latent space models (Li et al., 2020). Specifically, 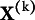 denotes the 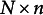 matrix of data in fold 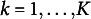 , and 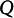 denotes the number of observed elements in 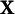 . Then, the data in fold 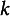 , 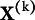 , are obtained by randomly selecting 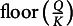 elements 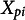 from the full data matrix 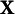 without replacement, and assigning these to elements 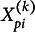 in 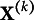 . The elements of 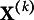 that have no value from 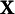 assigned are replaced by missing values. As such, 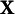 is partitioned in 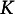 subsamples. If 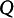 is not a multiple of 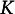 , the elements from 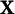 that are yet unselected are assigned to the first 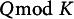 folds.

Next, the LSIRM is fit 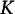 times to the data matrix 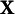 , each time leaving out the data from fold 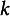 by assuming these data to be Missing Completely at Random (Rubin, 1976). Finally, for each of the 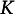 results obtained, we determine the predictive accuracy of the LSIRM in predicting the data from fold 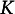 . Specifically, we consider three performance metrics.

#### 2.5.1 Unnormalized classification error

The first metric is what we refer to as the unnormalized classification error ( 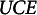 ):
(14)

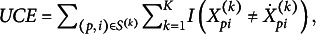

 where 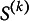 is the set containing all 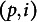 combinations for which 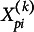 is observed in 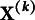 and 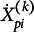 is the model predicted score (0 or 1) for 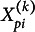 , that is,
(15)

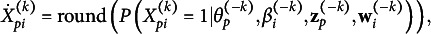

 where the parameters contain superscript 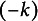 to denote that the data in 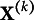 were not used in its estimation. Note that 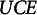 is inversely related to the classification accuracy, which is generally defined as the overall proportion of scores correctly classified as 0 or 1. We focus on classifications error so that smaller values indicate better-performing models, which is better in line with the residual sum of squares that we introduce later (which is naturally smaller for better-performing models). In addition, while the classification accuracy is commonly normalized, we focus on the unnormalized metric to increase the range of the metric and to be better in line with log-likelihood-based metrics, which are typically unnormalized (at least in psychometrics). The disadvantage of using unnormalized metrics is that its exact value is harder to interpret compared with, for example, prediction accuracies. However, prediction accuracies can be very similar across models due to their narrow range and upper bound (which is not necessarily 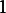 ; see Bergner et al., 2022), and can therefore also be challenging to interpret.

#### 2.5.2 Unnormalized ROC error

The next fit metric proposed is based on the area under the receiver operator curve (ROC). An ROC gives the relation among the true positive rates and the false positive rates of a model across different thresholds in a classification. For a perfect classification, the area under this curve is equal to 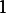 , indicating that the true positive rate is 1 and the false positive rate is 0. In the present study, we use the area under the ROC curve to see how well the data in a given fold are predicted using the LSIRM as estimated on the remaining folds. As discussed above, we focus on unnormalized metrics for which lower values indicate better model performance; therefore, we focus on the unnormalized ROC error ( 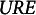 ):
(16)

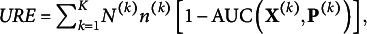

 where 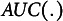 is the function that determines the area under the ROC curve and 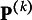 is an 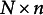 matrix containing the model predictions for fold 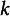 in elements 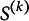 of that matrix. Similarly to 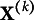 , the elements of 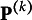 that are not in 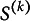 are set to missing.

#### 2.5.3 Residual sum of squares

Finally, we use a metric based on the residual sum of squares ( 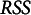 ) in the prediction of 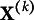 by 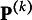 . That is,
(17)

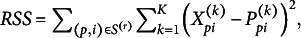

 where 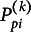 is element 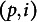 from matrix 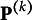 . For the 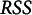 , it also holds that lower values indicate a better-performing model.

### 2.6 Likelihood optimization

We implemented the methods above in the 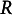 package *LSMjml*, which is available from CRAN and from www.dylanmolenaar.nl. We use a gradient ascent algorithm to maximize the likelihood in Equation (5) for pJML and in Equation (7) for cJML with 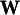 subject to the echelon structure discussed above. For pJML, parameters 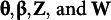 are updated sequentially by holding the other parameters constant. The likelihood is penalized as shown in Equation (5), with 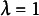 as discussed above. For cJML, parameter pairs 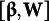 and 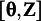 are updated alternatingly. The constraints in Equation (6) are introduced by gradient projection (Chen et al., 2019; Nocedal & Wright, 2006, p. 485) in which the parameter values are transformed to comply to Equation (6) after each parameter vector update (if necessary). As both JML procedures are full information, missing data can be accommodated by assuming MAR and summing the log-likelihood over all available data points.

Starting values are based on a preliminary exploratory item factor analysis with 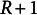 factors in which the first factor has equal factor loadings for all items and the factor loadings of the remaining 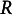 factors follow an echelon structure. Cai (2010) proposes a similar strategy for multidimensional item factor analysis. However, like Bergner et al. (2022), we found that, in general, random initialization performs equally well. See Algorithms 1 and 2 below for a description of, respectively, the pJML and cJML procedures using pseudocode.

**Figure:**
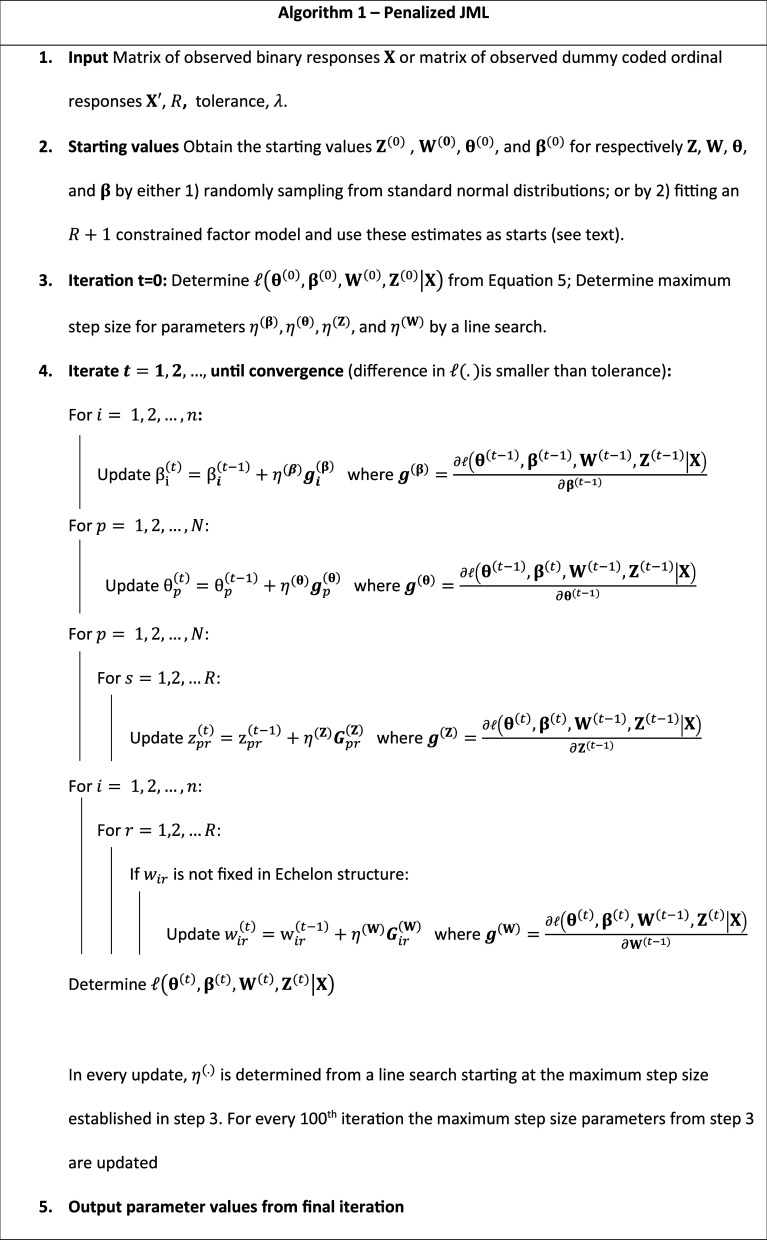

**Figure:**
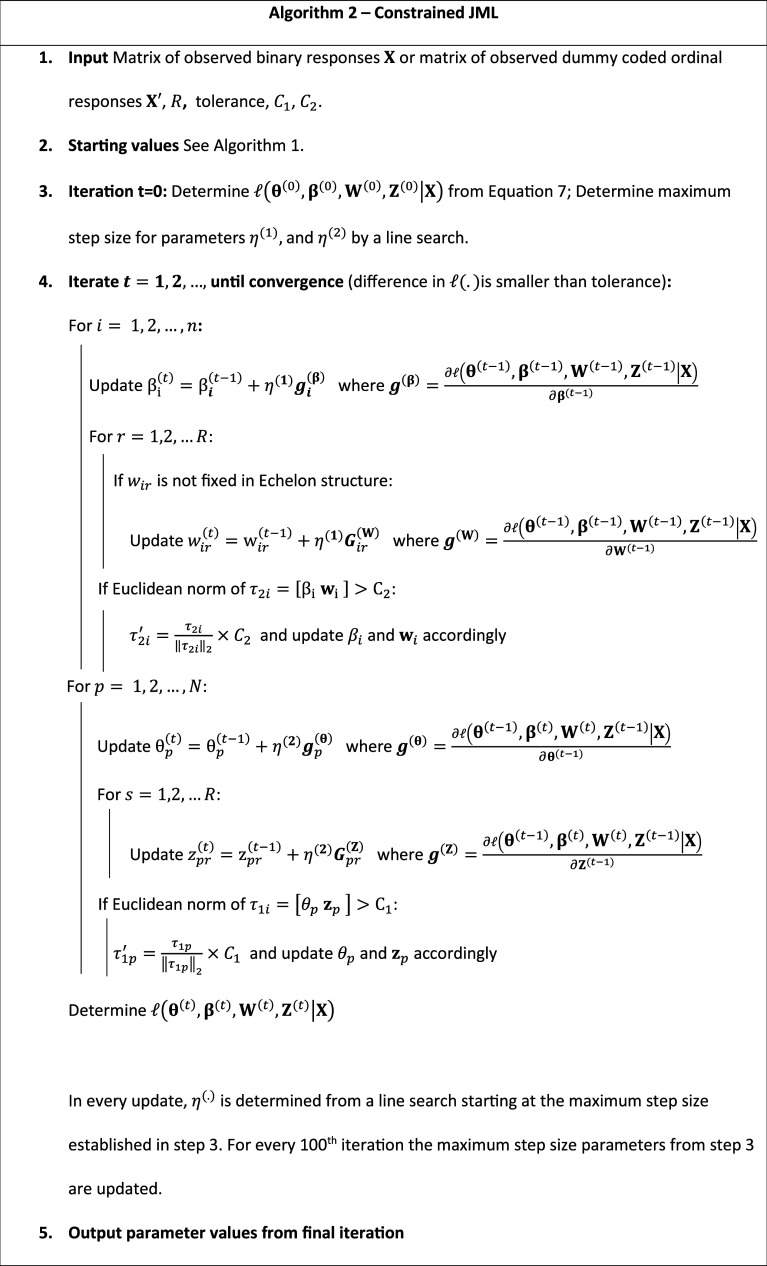

### 2.7 Ordered categorical data

With the methods above in place, a generalization to ordered categorical data is straightforward. That is, the LSIRM is formulated as a sequential item IRT model (Tutz, 1990) as follows:
(18)

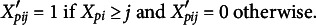

 where 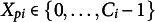 are the ordered categorical item responses and 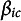 is the effect of item category 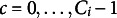 , which are ordered as follows: 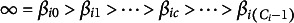 . Note that 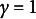 as discussed above.

This ordinal LSIRM can readily be fit using the methodology discussed above by recoding the 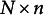 data matrix 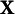 with order categorical elements 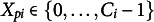 into an 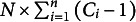 matrix of cumulative binary dummy coded variables 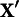 with elements 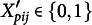 , for 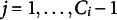 . Specifically,
(19)

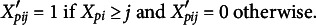



For instance, if 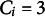 , this results in 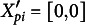 for 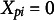 , 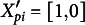 for 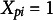 , and 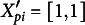 for 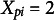 . Now, by treating the variables 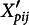 as dummy items in the binary LSIRM in Equation (18), and by fixing 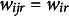 for dummy items that correspond to the same original item, fitting the cJML or pJML models to these dummy items will be equivalent to fitting Equation (18) to the original data 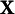 . The estimates of 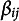 on 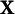 ’ will correspond to those of 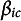 in Equation (18) obtained on 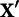 . Note that by using the approach above, the number of categories for the item responses is allowed to differ across items.

## 3 Simulation studies

### 3.1 Design

In this simulation study, we focus on parameter recovery of JML approaches proposed in this article. We focus on an LSIRM with 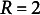 , as this is arguably the model that is used in practice most. In addition, we focus on binary data to be able to compare the results to the existing MCMC approach, which is only available for binary data. Moreover, the binary model underlies the ordinal model, as discussed above; therefore, the results will apply to the ordinal case as well. We simulate the data using the parameterization in Equations (1) and (2) (including the 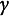 parameter). Note that the JML models do not include 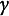 as a free parameter as explained above; however, we do simulate data using 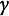 to manipulate the effect of the latent space on the data and to demonstrate that 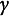 is indeed absorbed in the 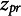 and 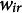 JML estimates.

We consider two sample sizes: 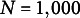 to reflect a more practical setting and 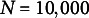 to study the large sample behavior of the model estimates. We fully cross these sample size conditions with 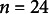 (practical setting) and 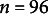 (large sample). Below, we discuss the true values for the parameters, which are inspired by or equal to the MCMC estimates of the deductive reasoning data analyzed in the illustration section below. Specifically, true values for 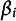 are between 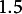 and 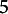 and are identical to the deductive reasoning data estimates. In addition, 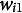 are decreasing from 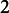 to 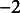 in equally sized steps and 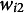 are between −2 and 2 and randomly assigned to the different items. For the conditions with 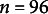 , the item parameters from the conditions with 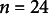 are repeated four times. For the person parameters, 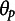 is specified to follow a normal distribution with mean 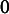 and variance 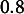 , and 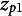 and 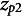 are specified to follow a normal distribution with mean 0 and variance, respectively, 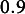 and 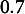 . The correlation between 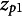 and 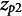 was specified to be equal to 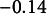 . Finally, 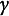 is equal to 1.7. As the above values are based on the MCMC results from the deductive reasoning data, we also wanted to add a data condition where 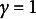 to establish how parameter recovery is affected by this parameter. All other parameters in this condition are the same as above. We use 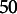 replications for each condition

To the data from the different conditions, we fit the LSIRM with 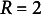 using both pJML and cJML as discussed above. We also fit the LSIRM using MCMC estimation in the R package *lsirm12pl* (Go et al., 2023). We did so to have a benchmark approach available to compare JML results to. That is, we want to demonstrate that parameter estimates are highly comparable across the different fitting methods after appropriate rotation. The aim of this study is not to show superiority of one method over the other. We used all default settings of *lsirm12pl*. That is, we relied on 15,000 posterior samples of which 2,500 are burn-in and which is thinned by 5. In addition, the default priors of the package are a standard normal prior for 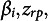 and 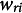 , a normal prior with mean 0 and variance 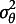 for 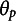 , a normal prior with mean 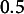 and variance 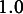 for 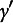 , and an inverse-gamma prior with scale and shape 0.001 for 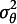 .

### 3.2 Results

As the results do not differ importantly across the different dimensions of 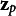 and 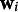 , in the below, we focus on 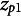 and 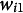 . In addition, due to space limitations, in the main text, we mostly provide graphical and verbal displays of the results, but we will make references to tables that can be found in the Supplementary Material.

#### 3.2.1 Parameter recovery

**Figure 2:**
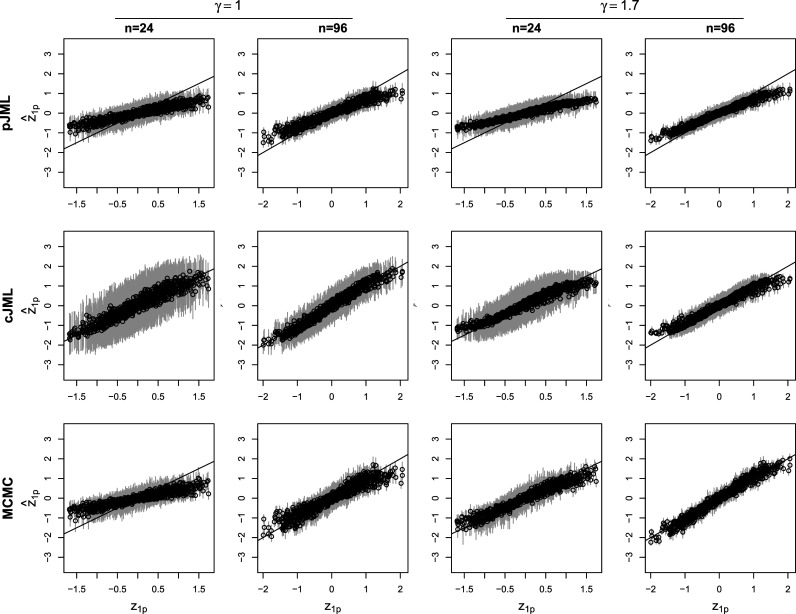
Plot of the true values of 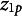 (*x*-axis) and the mean estimated values (*y*-axis) for penalized joint maximum likelihood (pJML), constrained joint maximum likelihood (cJML), and Markov Chain Monte Carlo for 24 and 96 items in the conditions 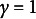 and 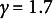 . In addition, 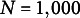 in all plots and the gray vertical lines indicate the range of the estimates within one standard deviation from the mean. Note that the pJML and cJML estimates are divided by the true value of 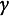 (see the text).

**Figure 3:**
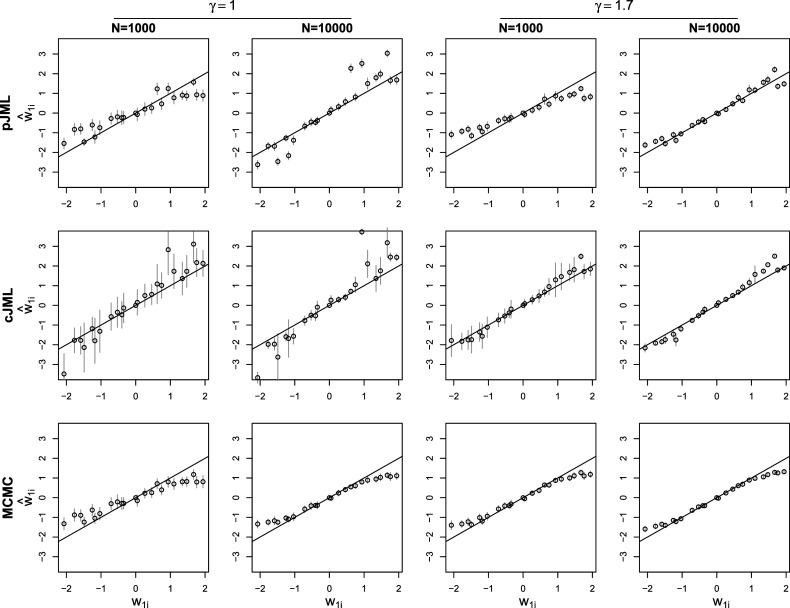
Plot of the true values of 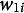 (*x*-axis) and the mean estimated values (*y*-axis) for penalized joint maximum likelihood (pJML), constrained joint maximum likelihood (cJML), and Markov Chain Monte Carlo for 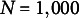 and 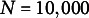 in the conditions 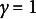 and 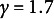 . In addition, 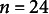 in all plots and the gray vertical lines indicate the range of the estimates within one standard deviation from the mean. Note that the pJML and cJML estimates are divided by the true value of 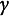 (see the text).

Figure 2 contains the average estimates and standard deviations across replications for 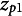 in the cases of 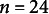 and 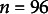 in the 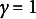 and 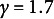 conditions for 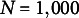 . In addition, similarly, Figure 3 contains the average estimates and standard deviations across replications for 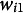 in the cases of 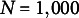 and 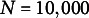 in the 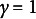 and 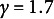 conditions for 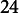 items. In the figures, the true values are the plots on the *x*-axis and the mean estimates on the *y*-axis. For the pJML and cJML estimates in the condition 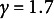 , the estimates are divided by 1.7, as the 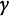 parameter is absorbed in these estimates (as it is not a free parameter in the JML approaches, see above). As can be seen, pJML and MCMC estimates tend to be unbiased up to a shrinkage effect that decreases for increasing 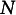 and 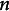 . For cJML, there is no shrinkage effect noticeable, which is understandable given the constraints used in this approach (Equations (8) and (9)), which operate as uniform priors. Shrinkage of the MCMC estimates diminishes at a higher rate for increasing 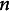 or 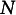 and 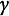 compared to pJML especially for 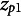 .

In Supplementary Table A1, the mean absolute bias (MAB), the variance (VAR), and the mean squared error (MSE) of the estimates for person parameters 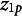 and 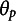 averaged over persons are reported. To separate the shrinkage effects from these statistics, for each parameter, the estimates are scaled to have a standard deviation equal to that of the true parameters. The recovery of the standard deviation of the estimates is studied separately later.

The most important result is that for all three methods, the MAB, VAR, and MSE decrease for 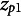 and 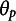 for an increasing number of items, 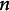 . In addition, pJML performs comparable to MCMC especially for larger 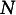 and 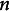 . For cJML, the VAR and MSE are slightly higher compared with cJML and MCMC, particularly in conditions with smaller 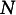 and 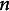 .

Supplementary Table A2 contains the MSE, VAR, and MAB for item parameters 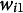 and 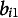 averaged over items. For all three methods, these statistics decrease for both 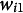 and 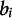 for an increasing sample size, 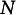 . One exception is for the estimates of 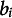 for pJML in the 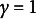 condition where the VAR increases slightly. Similar as for the person parameters, for 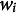 , pJML performs comparable to MCMC especially for larger 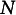 and 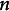 . For cJML, the VAR and MSE are slightly higher compared with cJML and MCMC particularly in conditions with smaller 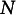 and 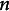 . For 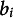 , results are generally somewhat better for MCMC in the case of smaller 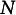 , 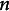 , and 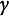 .

Next, we focus on accuracy of the standard deviation of the estimates. As the variance of these standard deviations is very small, we only report the mean bias in Supplementary Table A3. Previously, we focused on the MAB to prevent biasing effects from canceling out across items or persons. However, here, the mean bias is more interesting as it gives an indication of the direction of the effect. That is, a mean bias smaller than 0 indicates shrinkage of the parameter estimates, whereas a mean bias larger than 0 indicates increased parameter variability. In addition, as we are not aggregating over persons or items, effects cannot cancel out.

As the standard deviations of the parameters are not explicit model parameters for pJML and cJML, and these parameters are fixed for MCMC (except for 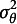 ), the mean bias of the standard deviations has been determined by focussing on the theoretically expected standard deviation. For MCMC, the expected standard deviation is the standard deviations of the true values used to simulate the data (see above). For pJML and cJML, the expected value of 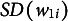 and 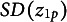 are, respectively, 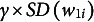 and 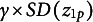 as 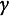 is expected to be absorbed in 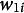 and 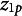 as discussed above. For 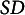 ( 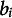 ) and 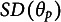 , the expected values are the standard deviations of the true values used to simulate the data. The results show that, as was already concluded from Figures 2 and 3, shrinkage effects are present for pJML and MCMC with slightly negative biases that decrease for increasing 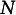 and 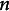 . This decrease generally follows a higher rate for MCMC compared with pJML. For cJML, the estimates are mostly associated with increased variability with positive biases that decrease for increasing 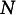 and 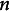 .

#### 3.2.2 Estimation time

**Figure 4:**
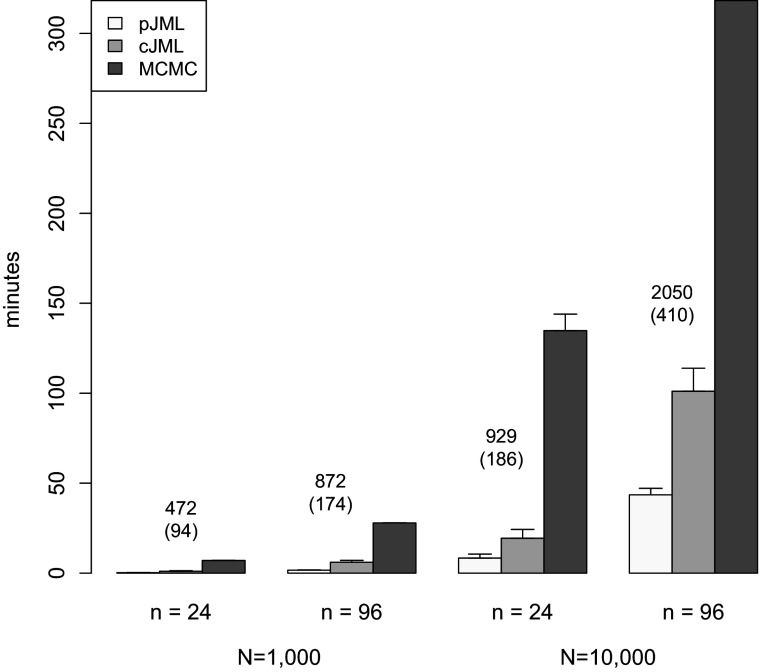
Bar plot of the mean estimation time in minutes for penalized joint maximum likelihood (pJML), constrained joint maximum likelihood, and Markov Chain Monte Carlo for the different conditions and 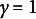 . The error bars indicate one standard deviation. The numbers indicate the rounded number of samples you can take from the posterior parameters in the average time pJML took to converge (after thinning in brackets).

Figure 4 contains a bar plot of the mean estimation time of the different approaches for 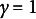 in minutes. For 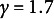 , pJML and cJML are even faster, while MCMC is about as fast (see Supplementary Table A4). It can be concluded that pJML is the fastest, and MCMC is the slowest. These averages are just given as an indication; the comparison between cJML/pJML and MCMC may not be fully fair as the settings for the MCMC routine (e.g., number of samples) may be tuned in specific conditions so that the average times shown in the figure are an overestimate. However, looking at the number of samples you can take from the posterior parameters in the average time pJML took to converge (also in the figure), it is clear that the JML-based approaches require a substantial smaller amount of time. As discussed, this brings possibilities for establishing model fit, which are discussed below.

#### 3.2.3 Cross-validation

We conducted additional simulations to test the appropriateness of our proposed 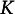 -folds cross-validation procedure. The full description of this study and the results can be found in Appendix B of the Supplementary Material. The main results indicate that cross-validation with the UCE, URE, and RSS metrics as proposed above can distinguish well between the models for 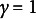 , irrespective of the number of items, the number of persons, or the number of folds considered. For the smaller data scenario ( 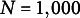 , 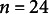 ), the 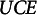 has difficulties, correctly identifying the 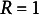 model for five folds (true positive rate of 0 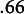 ). However, using 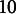 folds, the true positive rate increases to 0.78, which is more acceptable. For 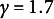 , all true positives were at least 0.98, with one exception of a true positive of 0.86 for the 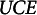 when 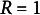 and 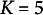 .

It should be noted that our cross-validation procedure tends to overselect, and not underselect. That is, although, in general, the correct model is picked most of the times, if an incorrect model is being selected, it is always a model with a larger 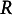 than the true model. This is a known property of cross-validation in general (see Li et al., 2020), but it should be kept in mind when using cross-validation for dimensionality selection.

### 3.3 Conclusion

In general, our cJML and pJML estimation approaches perform as intended. The parameter recovery is comparable to that of MCMC with some minor differences in specific conditions due to differences in the priors and restrictions imposed. The cJML estimates are generally more variable because of the constraints in this approach being the least restrictive among the three approaches. However, it should be noted that in practice, 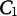 and 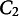 may be chosen by cross-validation, which may decrease parameter variability. The pJML approach is generally faster than cJML, with MCMC being slower than the JML approaches. Finally, the cross-validation procedure works well in selecting the number of dimensions of the latent space.

## 4 Illustration 1: dichotomous data

### 4.1 Data

In this section, we analyze the scores of 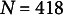 children to 24 items from a Piagetian syllogistic deductive reasoning test (see Spiel et al., 2001). These data have previously been analyzed using an MCMC LSIRM by Jeon et al. (2021). In the present application, we use these data to illustrate the similarities between the results of JML and MCMC after appropriate rotation. In addition, Jeon et al. tested the fit of an 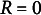 LSIRM against an 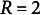 LSIRM using the spike and slab prior approach discussed above. Here, we verify these results using the present cross-validation approach with 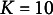 and extend these results by also considering 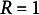 and 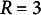 . In addition, we explore the results of the 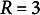 model. Models are fit using the same methods and settings as discussed for Simulation Study 1.

### 4.2 Results

We first consider the LSIRM with 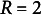 as studied by Jeon et al. (2021) in an MCMC framework. After the rotation of the MCMC results to an echelon structure, correlations among the parameter estimates across the different approaches are close to 1.0 (i.e., at least 0.989 for the MCMC—JML parameter estimate correlations) or practically 1.0 (i.e., at least 0.999 for the pJML and cJML parameter estimate correlations). We, therefore, only consider the pJML results below.

**Figure 5:**
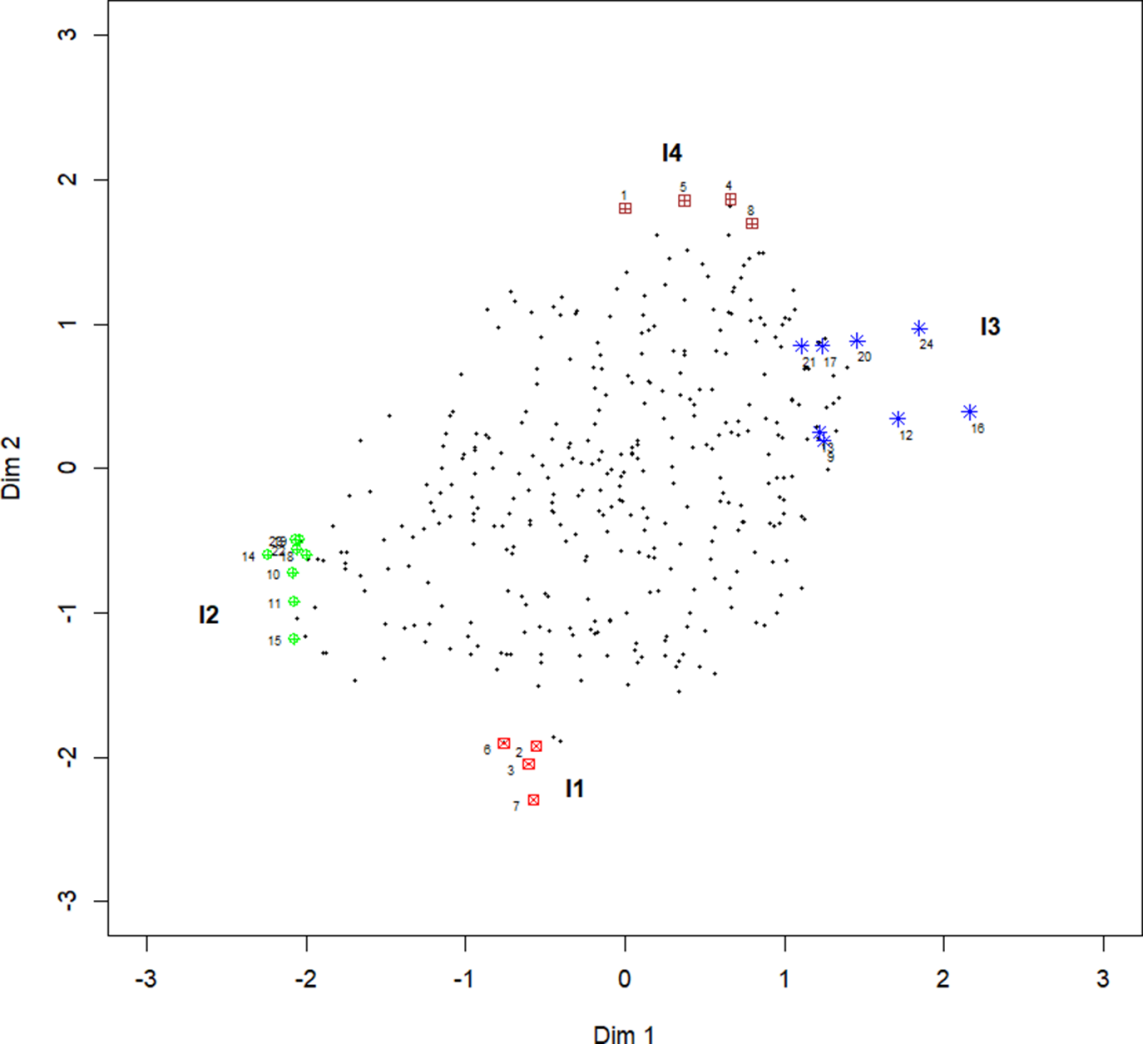
Plot of the estimates of vector 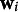 from an 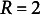 model for all items. I1–I4 are the item groups identified by Jeon et al. (2021).

Table 1 contains the results of the cross-validation for models with 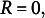 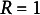 , 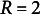 , 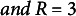 . As can be seen, the 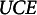 and 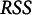 select the 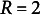 model to be the best fitting, whereas the 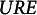 favors the 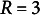 model. As Jeon et al. (2021) considered an 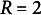 model, we here also explore if an 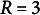 model will give some additional insights. First, in Figure 5, the 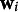 and 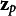 estimates are plotted for an 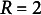 LSIRM. The figure is highly comparable to Figure 8a of Jeon et al., up to a difference in orientation due to the differences in rotation between pJML and MCMC. Following Jeon et al., we label the four item clusters that are observed as “I1” to “I4” in the figure. These are interpreted as, respectively, concrete complex inference items, abstract and counterfactual logical fallacy items, bi-conditional items, and complex algebra items.

**Table 1:** Model fit indices as based on a 10-fold cross-validation.

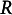	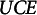	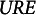	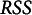
0	3,209	2,424	2,049
1	2,536	1,571	1,729
2	**2,403**	1,416	**1,688**
3	2,426	**1,364**	1,696

*Note*: For each metric, the smallest value is in boldface. 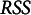 , residual sum of squares; 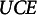 , unnormalized classification error; 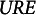 , unnormalized ROC error.

**Figure 6:**
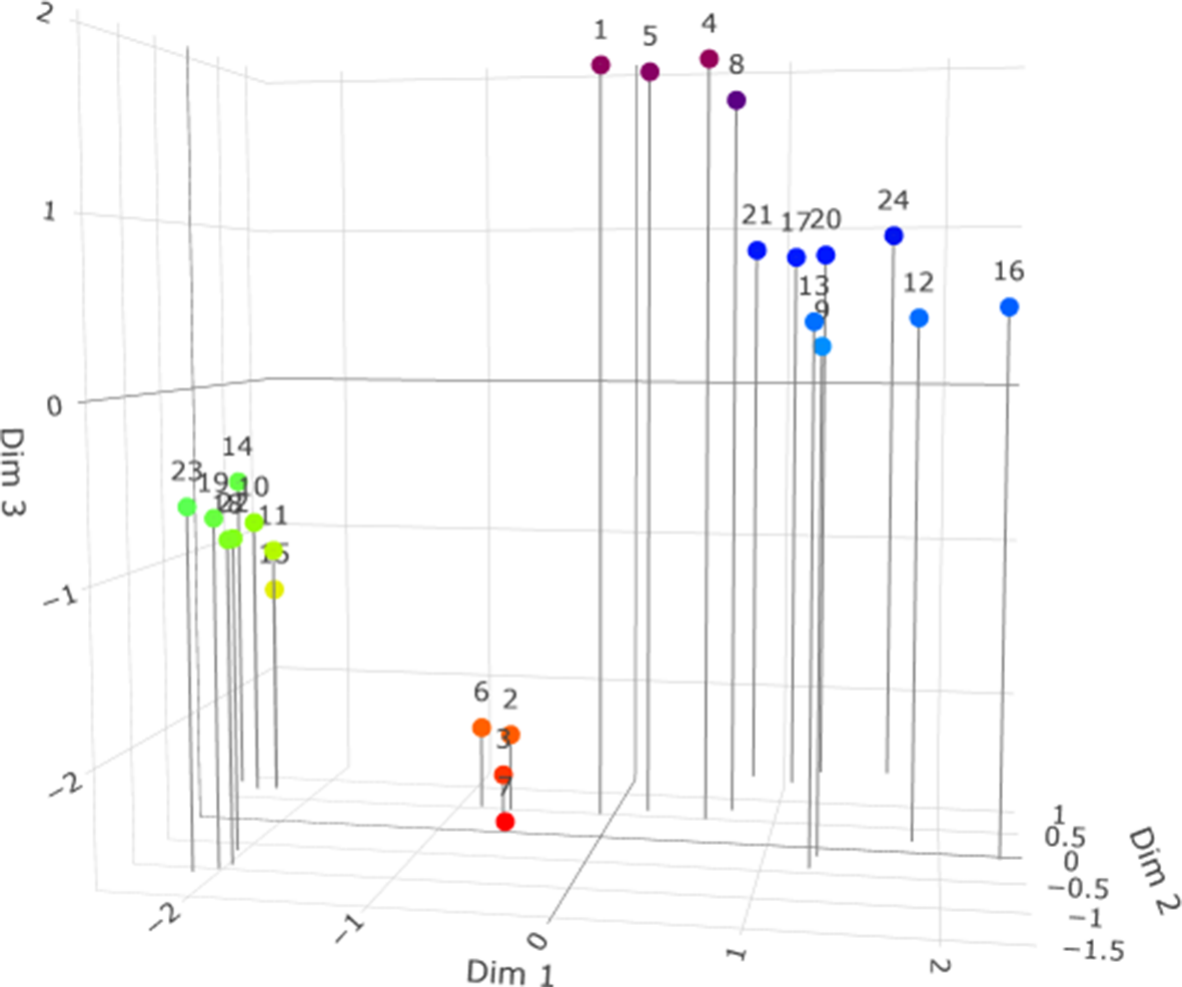
Plot of the estimates of vector 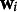 from an 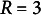 model for all items. The view has been adjusted to demonstrate correspondence with the results for the 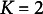 model, which lacks dimension 1 (see the text).

**Figure 7:**
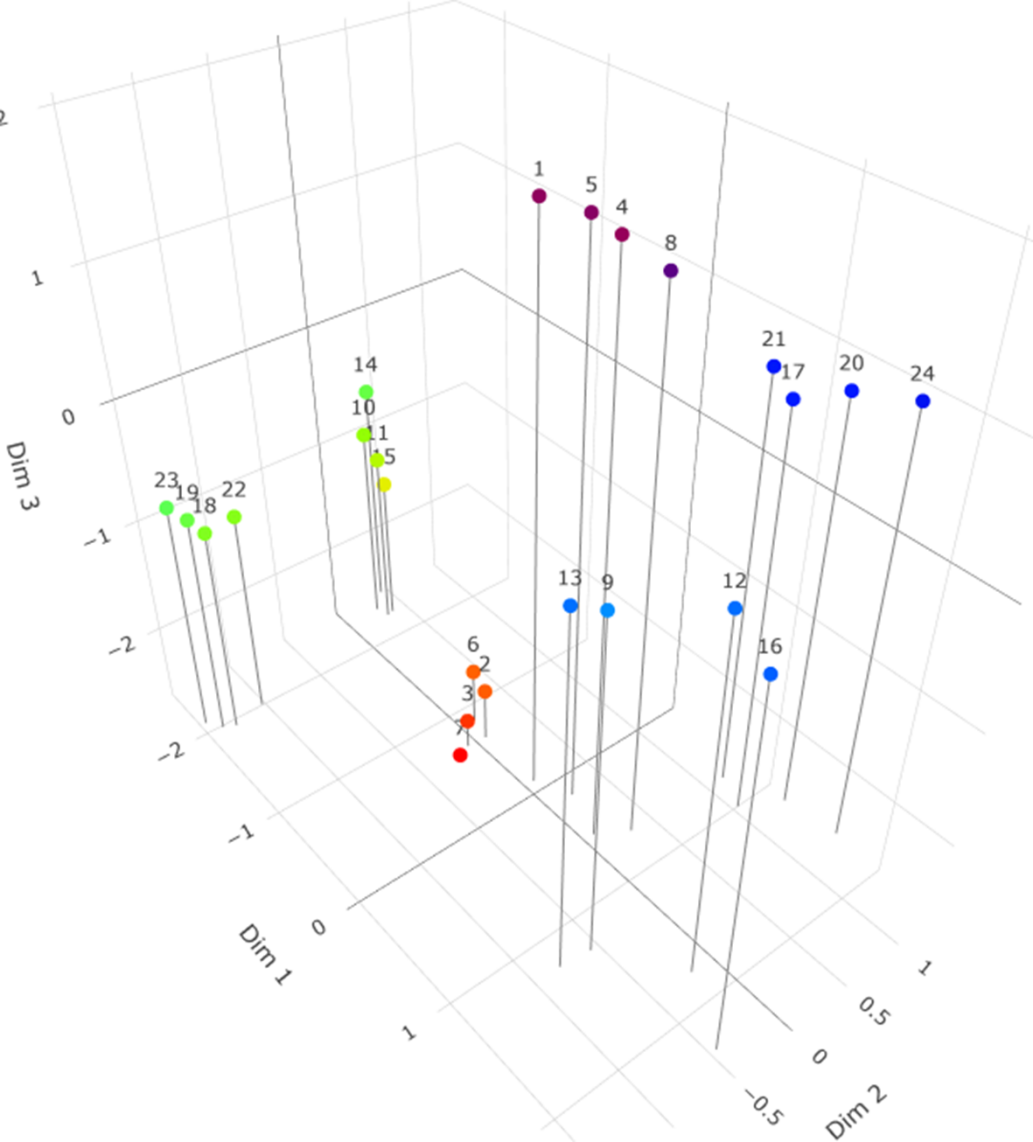
Plot of the estimates of vector 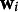 from an 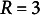 model for all items. The view has been chosen to demonstrate the differences across dimension 1.

**Figure 8:**
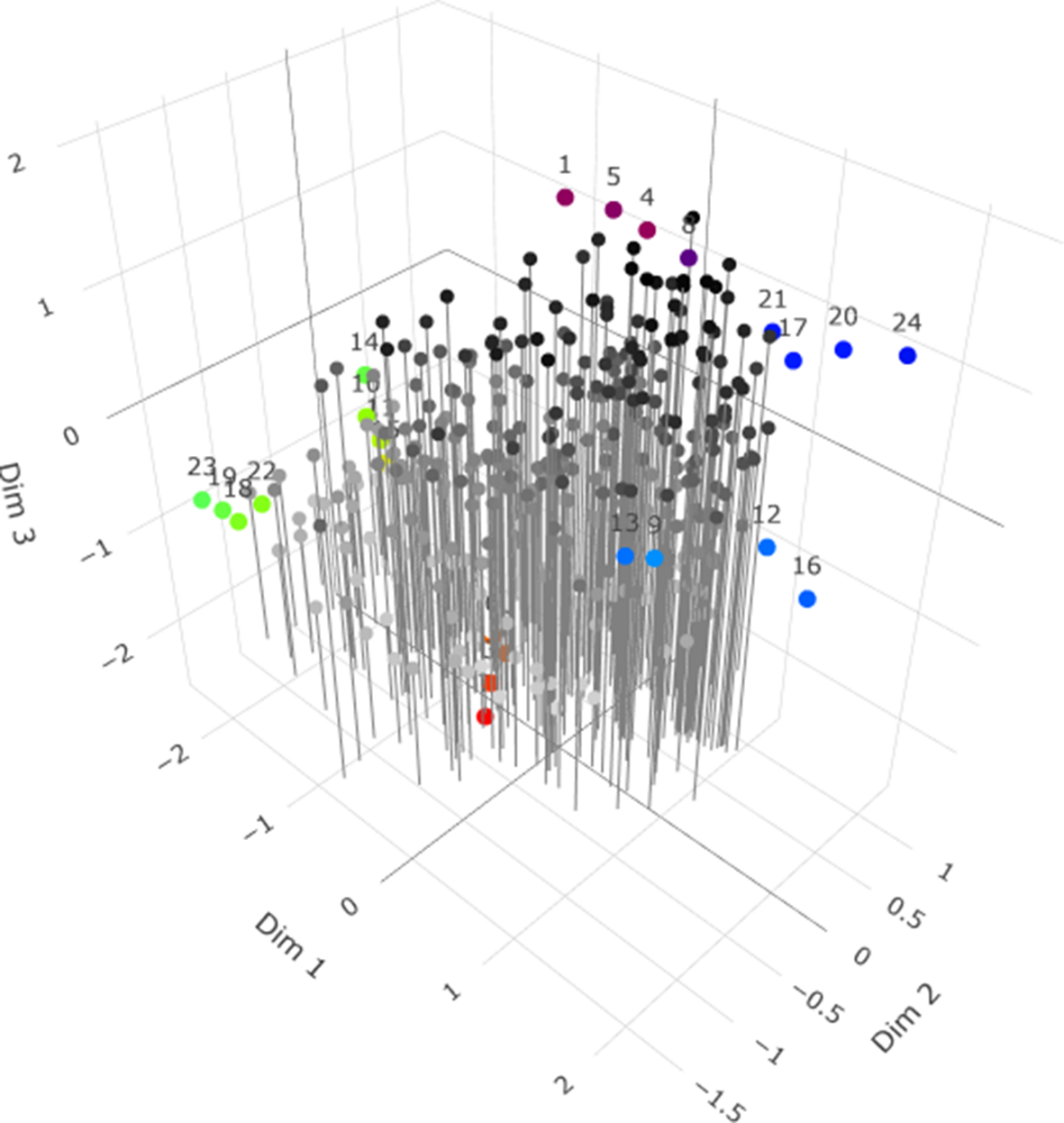
Same as Figure 7, but with the estimates of 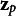 added.

Next, we fit an 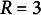 LSIRM. Figure 6 contains a three-dimensional plot of the estimates of 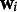 from this model. We first focus on an orientation of the view of the plot in which variability on dimension 1 ( 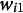 ) is mostly masked to show the correspondence with the 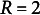 plot in Figure 5. It is important to note that by using the echelon restrictions in the way illustrated in Equation (10), the first dimension from an 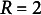 model corresponds to the second dimension in an 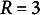 model. Therefore, we masked dimension 1 in Figure 6 to show the correspondence to Figure 5. As these figures indeed correspond, we now demonstrate the differences on the first dimension in Figure 7. As can be seen, an additional item cluster seems to arise splitting cluster I2 into l2a and l2b where I2a are items 10, 11, 14, and 15, and I2b are items 18, 19, 22, and 23. In Figure 8, we next add the estimates of 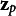 to the three-dimensional plot. This figure illustrates that persons differ in their proximity to the different item clusters, with the I3 items (blue) being more isolated, for instance, compared with the I1 items (red).

### 4.3 Conclusion

The main purpose of this real data analysis was to illustrate our JML-based approach on real data. A cross-validation indicated that an 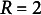 model seems, indeed, appropriate for the deductive reasoning task analyses by Jeon et al. (2021) using an MCMC approach. As results indicated that there may be variability on an additional dimension, we explored a three-dimensional model and found some differences among the items in the three-dimensional space.

## 5 Illustration 2: ordered categorical data

### 5.1 Data

In this section, we illustrate the application of the present methodology to the analysis of ordinal data. To this end, we analyze data from the Adjective Check List (ACL; Gough & Heilbrun, 1980). The original ACL consists of 300 adjectives for which respondents need to indicate on a 5-point scale to what degree the adjective describes the personality of the respondent. The item scores of 433 students on an adapted version of the ACL is available as part of the Mokken package in R (Van der Ark, 2007). Here, we focus on the Communality and the Dominance scale, which both consist of 10 items. We fit the ordinal LSIRM in Equation (18) to each scale separately using the cumulative binary dummy coding approach described above. We focus on 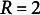 for ease of interpretation and visualization. The contraindicative items (indicated by an asterisk in the item name in the presentation of the results) are reversely coded. For the item “reliable” from the Communality scale, respondents did not use the lowest response category. Therefore, this item only has three threshold parameters, and all other items have four threshold parameters.

### 5.2 Results

**Figure 9:**
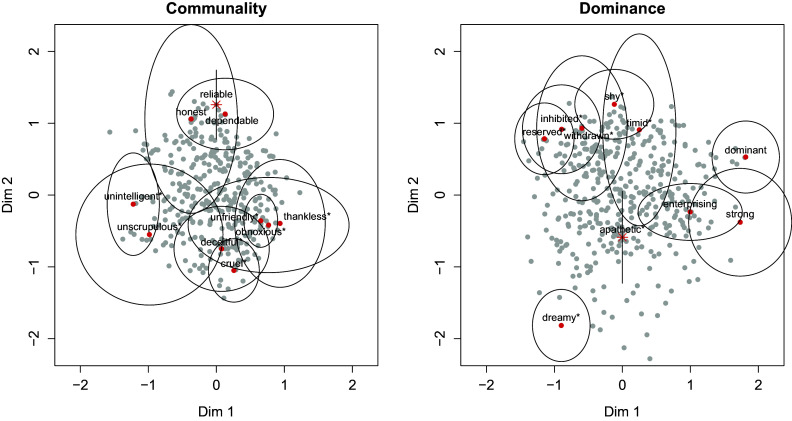
Graphical representation of the ordinal latent space item response model in application 2 for the Communality scale (left) and the Dominance scale (right) of the ACL. The red dots represent the item locations 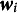 , and the gray dots represent the person locations 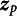 . The ellipses around the item locations give the range of the 99% confidence intervals. The location of the first item (“reliable” in Communality scale and “apathic” in the Dominance scale) is indicated by a star instead of a dot as 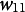 is fixed to 0 for this item, and a 99% confidence line is displayed for this item.

Item parameter estimates for 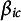 and 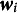 of the ordinal LSIRM can be found in Supplementary Table C1. In Figure 9, the latent space, including 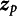 , is displayed for the two scales. For the item positions 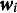 , the figure contains 99% confidence ellipses based on bootstrapped standard errors using 1,000 samples. Even though the scales are intended to be unidimensional, as can be seen from the figure, for both scales, the items seem to meaningfully cluster. For instance, for the Communality scale, omitting the “unscrupulous*” and “honest” items that have relatively large standard errors, three clusters arise: the “unintelligent*” item on the on side, which is apart from all the other items; the “reliable”–“dependable” cluster; and a cluster with among others “cruel*” and “unfriendly*.” Items within a cluster are close to each other while being further away from the other clusters. For the Dominance scale, something similar is observed, omitting the “timid*” item, which has a relatively large standard error, “dreamy*” stands out as it is apart from most of the items, while “dominant,” “strong,” and “enterprising” form a cluster, and the other items form a separate cluster. “Apathic*” is separate but in the middle of the three clusters.

For the person positions, respondents are scattered across the clusters, with some persons have an estimate closer to, for example, the “reserved*–withdrawn*” item cluster than to other items. Respondents near the origin of the coordinate system (0,0) score high or low on all items (which could be inferred from their 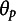 estimate).

### 5.3 Conclusion

We demonstrated the viability of the ordinal LSIRM on a real dataset. In practice, results like the above can be used to make profiles. For instance, using the adjectives check list above, in job applicant selection, one may want to aim for respondents close to the “enterprising” and “strong” items, but also to the shy* and timid* items, while not necessarily close to the “dreamy*” item. Using the present approach such distances between persons and items can readily be interpreted. Other statistical methods like factor analysis are valuable but serve different purposes compared with the latent space approach adopted here. That is, factor analysis parameters cannot be interpreted in terms of item–person distances as in factor analysis triangular inequality is violated. For a more elaborate discussion on the differences, see Jeon et al. (2021).

## 6 Discussion

In this study, we proposed regularized JML estimation to fit the LSIRM to data. A key advantage is that the estimation is fast, which hopefully facilitates the use of these models by the applied researcher. Owing to its fast nature, model selection can be conducted using 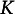 -folds cross-validation, which is valuable as such model selection tools are still relatively limited in the LSIRM literature.

These advantages come at the cost of reduced information about the posterior parameter distribution. That is, the MCMC approach results in estimates of the full posteriors parameter distributions, while, as discussed, the estimates in our approach can be conceived as MAP estimates (e.g., Swaminathan & Gifford, 1985). Our approach does not give such detailed information about the shape of the posterior parameter distributions. In the illustration section, we bootstrapped the standard errors, but this increases the computational burden. The observed and expected information matrices may be useful in obtaining uncertainty measures; however, for our approach, the theoretical properties of these matrices are strictly unknow. At best, they give a local approximation if the posterior is normal near its mode. As MCMC can account for any form of the parameter distribution in principle, MCMC is superior in its measures of parameter uncertainty. Therefore, as noted earlier, we do not view our approach as a replacement for existing LSIRM modeling tools, but rather as an extension. For example, when uncertainty measures are of primary importance, MCMC estimation remains preferable. Conversely, if the main goal is to visualize latent space positions or determine the dimensions of the latent space, our JML-based approaches are recommended.

As discussed in this article, traditional JML estimation as proposed by Birnbaum (1968) is known to be asymptotically inconsistent (e.g., Haberman, 1977). For a multidimensional unstructured two-parameter logistic model, Chen et al. (2019) proofed JML to be consistent in the double asymptotic sense under appropriate constraints. As mentioned, we did not study such theoretical consistency for our approach. However, the simulation results suggest that parameter bias decreases for increasing number of items and sample size. Moreover, theoretical consistency was not our main objective in this study. That is, we studied JML as a less time-consuming complement to the existing MCMC procedures but with comparable results. In that respect, our approach performs as desired. It should be noted, however, that our implementation of the constrained JML approach uses the results from Chen et al. (2019), which are known to be asymptotically consistent in the average sense (the overall MSE approaches 0). For entry-wise consistency (i.e., MSE approaches 0 for each parameter), additional post processing steps are needed (see Chen and Li, 2024). Here, we did not consider such post processing, but we note that our approach is equally amenable to such a treatment.

## Supporting information

10.1017/psy.2025.10068.sm001 Molenaar and Jeon supplementary material Molenaar and Jeon supplementary material

## Supplementary material.

To view supplementary material for this article, please visit http://doi.org/10.1017/psy.2025.10068.
