# Supplementary material for: Regularized Joint Maximum Likelihood Estimation of Latent Space Item Response Models
Source: Psychometrika. 2026 Jan 9;91(1):335–59. doi: 10.1017/psy.2025.10068 (PMC13121840; doi:10.1017/psy.2025.10068)
Supplement: Molenaar and Jeon supplementary material [file S0033312325100689sup001.docx]

**Appendix A:**

**Tables Accompanying the Results of the Simulation Study**

**Table A1.**

*The Mean Absolute Bias (MAB), variance, and Mean Squared Error (MSE) for* $z_{1p}$ *and* $\theta_{p}$ *aggregated over* $p$ *for the different conditions of the simulation study*

| $\gamma$ | $N$ | $n$ | Method |  | $z_{p1}$ | | |  | $\theta_{p}$ | | |
| --- | --- | --- | --- | --- | --- | --- | --- | --- | --- | --- | --- |
|  |  |  |  |  | *MAB* | *VAR* | *MSE* |  | *MAB* | *VAR* | *MSE* |
| $1$ | $1,000$ | 24 | pJML |  | 0.52 | 0.32 | 0.42 |  | 0.42 | 0.20 | 0.28 |
|  |  |  | cJML |  | 0.54 | 0.34 | 0.46 |  | 0.48 | 0.25 | 0.35 |
|  |  |  | MCMC |  | 0.51 | 0.31 | 0.42 |  | 0.42 | 0.19 | 0.28 |
|  |  | 96 | pJML |  | 0.37 | 0.17 | 0.22 |  | 0.36 | 0.09 | 0.20 |
|  |  |  | cJML |  | 0.39 | 0.21 | 0.25 |  | 0.33 | 0.15 | 0.17 |
|  |  |  | MCMC |  | 0.37 | 0.16 | 0.22 |  | 0.32 | 0.08 | 0.15 |
|  | $10,000$ | 24 | pJML |  | 0.50 | 0.30 | 0.39 |  | 0.42 | 0.20 | 0.27 |
|  |  |  | cJML |  | 0.52 | 0.33 | 0.43 |  | 0.47 | 0.24 | 0.34 |
|  |  |  | MCMC |  | 0.50 | 0.29 | 0.39 |  | 0.42 | 0.20 | 0.27 |
|  |  | 96 | pJML |  | 0.35 | 0.16 | 0.20 |  | 0.27 | 0.08 | 0.11 |
|  |  |  | cJML |  | 0.37 | 0.19 | 0.22 |  | 0.30 | 0.13 | 0.15 |
|  |  |  | MCMC |  | 0.35 | 0.15 | 0.20 |  | 0.27 | 0.09 | 0.11 |
| $1.7$ | $1,000$ | 24 | pJML |  | 0.35 | 0.16 | 0.20 |  | 0.42 | 0.18 | 0.28 |
|  |  |  | cJML |  | 0.36 | 0.17 | 0.21 |  | 0.46 | 0.20 | 0.33 |
|  |  |  | MCMC |  | 0.36 | 0.17 | 0.20 |  | 0.42 | 0.19 | 0.28 |
|  |  | 96 | pJML |  | 0.22 | 0.06 | 0.08 |  | 0.35 | 0.07 | 0.18 |
|  |  |  | cJML |  | 0.23 | 0.06 | 0.08 |  | 0.42 | 0.07 | 0.24 |
|  |  |  | MCMC |  | 0.22 | 0.06 | 0.08 |  | 0.31 | 0.09 | 0.15 |
|  | $10,000$ | 24 | pJML |  | 0.35 | 0.16 | 0.19 |  | 0.42 | 0.17 | 0.27 |
|  |  |  | cJML |  | 0.36 | 0.17 | 0.20 |  | 0.46 | 0.19 | 0.33 |
|  |  |  | MCMC |  | 0.35 | 0.16 | 0.20 |  | 0.42 | 0.20 | 0.27 |
|  |  | 96 | pJML |  | 0.21 | 0.06 | 0.07 |  | 0.27 | 0.07 | 0.12 |
|  |  |  | cJML |  | 0.22 | 0.06 | 0.08 |  | 0.44 | 0.07 | 0.26 |
|  |  |  | MCMC |  | 0.21 | 0.05 | 0.07 |  | 0.27 | 0.08 | 0.11 |

**Table A2.**

*The Mean Absolute Bias (MAB), variance, and Mean Squared Error (MSE) for* $w_{1i}$ *and* $b_{i}$ *aggregated over* $p$ *for the different conditions of the simulation study*

| $\gamma$ | $N$ | $n$ | Method |  | $w_{i1}$ | | |  | $b_{i}$ | | |
| --- | --- | --- | --- | --- | --- | --- | --- | --- | --- | --- | --- |
|  |  |  |  |  | *MAB* | *VAR* | *MSE* |  | *MAB* | *VAR* | *MSE* |
| $1$ | $1,000$ | 24 | pJML |  | 0.44 | 0.14 | 0.31 |  | 0.59 | 0.04 | 0.48 |
|  |  |  | cJML |  | 0.49 | 0.28 | 0.40 |  | 1.11 | 0.38 | 1.66 |
|  |  |  | MCMC |  | 0.39 | 0.17 | 0.26 |  | 0.58 | 0.02 | 0.46 |
|  |  | 96 | pJML |  | 0.30 | 0.09 | 0.14 |  | 0.70 | 0.04 | 0.60 |
|  |  |  | cJML |  | 0.39 | 0.24 | 0.26 |  | 0.60 | 0.33 | 0.64 |
|  |  |  | MCMC |  | 0.29 | 0.10 | 0.14 |  | 0.58 | 0.02 | 0.44 |
|  | $10,000$ | 24 | pJML |  | 0.32 | 0.02 | 0.18 |  | 0.53 | 0.08 | 0.42 |
|  |  |  | cJML |  | 0.36 | 0.08 | 0.23 |  | 1.12 | 0.20 | 1.71 |
|  |  |  | MCMC |  | 0.20 | 0.04 | 0.07 |  | 0.26 | 0.01 | 0.12 |
|  |  | 96 | pJML |  | 0.16 | 0.02 | 0.04 |  | 0.27 | 0.04 | 0.12 |
|  |  |  | cJML |  | 0.21 | 0.06 | 0.09 |  | 0.51 | 0.14 | 0.52 |
|  |  |  | MCMC |  | 0.14 | 0.02 | 0.03 |  | 0.22 | 0.01 | 0.09 |
| $1.7$ | $1,000$ | 24 | pJML |  | 0.25 | 0.03 | 0.11 |  | 1.20 | 0.03 | 1.69 |
|  |  |  | cJML |  | 0.23 | 0.06 | 0.10 |  | 0.42 | 0.16 | 0.30 |
|  |  |  | MCMC |  | 0.20 | 0.04 | 0.07 |  | 0.60 | 0.03 | 0.55 |
|  |  | 96 | pJML |  | 0.16 | 0.02 | 0.04 |  | 1.02 | 0.03 | 1.20 |
|  |  |  | cJML |  | 0.18 | 0.05 | 0.06 |  | 0.45 | 0.21 | 0.30 |
|  |  |  | MCMC |  | 0.16 | 0.03 | 0.04 |  | 0.61 | 0.03 | 0.53 |
|  | $10,000$ | 24 | pJML |  | 0.19 | 0.01 | 0.06 |  | 0.40 | 0.02 | 0.23 |
|  |  |  | cJML |  | 0.14 | 0.01 | 0.04 |  | 0.39 | 0.02 | 0.25 |
|  |  |  | MCMC |  | 0.13 | 0.01 | 0.03 |  | 0.34 | 0.01 | 0.21 |
|  |  | 96 | pJML |  | 0.08 | 0.01 | 0.01 |  | 0.25 | 0.02 | 0.09 |
|  |  |  | cJML |  | 0.10 | 0.01 | 0.02 |  | 0.37 | 0.04 | 0.18 |
|  |  |  | MCMC |  | 0.09 | 0.01 | 0.01 |  | 0.26 | 0.01 | 0.14 |

**Table A3.**

*The Mean Bias for the standard deviation of the parameter estimates for the different conditions of the simulation study*

| $\gamma$ | $N$ | $n$ | Method |  | Mean Bias | | | |
| --- | --- | --- | --- | --- | --- | --- | --- | --- |
|  |  |  |  |  | $SD(z_{p1})$ | $SD(\theta_{p})$ | $SD(w_{i1})$ | $SD(b_{i})$ |
| $1$ | $1,000$ | 24 | pJML |  | -0.14 | -0.12 | -0.29 | -0.19 |
|  |  |  | cJML |  | 0.60 | 0.35 | 0.68 | 0.04 |
|  |  |  | MCMC |  | -0.17 | -0.25 | -0.41 | -0.03 |
|  |  | 96 | pJML |  | -0.12 | -0.07 | -0.26 | -0.15 |
|  |  |  | cJML |  | 0.29 | 0.17 | 0.28 | 0.13 |
|  |  |  | MCMC |  | 0.03 | -0.14 | -0.22 | -0.06 |
|  | $10,000$ | 24 | pJML |  | -0.11 | -0.09 | 0.46 | -0.37 |
|  |  |  | cJML |  | 0.56 | 0.36 | 0.75 | -0.05 |
|  |  |  | MCMC |  | -0.10 | -0.24 | -0.32 | 0.06 |
|  |  | 96 | pJML |  | -0.09 | -0.03 | 0.22 | -0.19 |
|  |  |  | cJML |  | 0.22 | 0.16 | 0.32 | 0.02 |
|  |  |  | MCMC |  | 0.07 | -0.11 | -0.12 | 0.00 |
| $1.7$ | $1,000$ | 24 | pJML |  | -0.46 | -0.12 | -0.78 | -0.13 |
|  |  |  | cJML |  | 0.12 | 0.18 | 0.30 | 0.08 |
|  |  |  | MCMC |  | 0.06 | -0.32 | -0.27 | 0.06 |
|  |  | 96 | pJML |  | -0.30 | -0.07 | -0.52 | -0.10 |
|  |  |  | cJML |  | -0.08 | 0.04 | 0.23 | 0.01 |
|  |  |  | MCMC |  | 0.12 | -0.12 | -0.08 | -0.03 |
|  | $10,000$ | 24 | pJML |  | -0.42 | -0.09 | -0.06 | -0.06 |
|  |  |  | cJML |  | 0.11 | 0.19 | 0.40 | -0.02 |
|  |  |  | MCMC |  | 0.08 | -0.26 | -0.20 | 0.12 |
|  |  | 96 | pJML |  | -0.27 | -0.04 | 0.07 | -0.10 |
|  |  |  | cJML |  | -0.09 | 0.05 | 0.30 | -0.08 |
|  |  |  | MCMC |  | 0.14 | -0.10 | 0.00 | 0.00 |

*Note*. As the standard deviations of the parameters are not estimated model parameters (exception: $SD(\theta_{p})$ for MCMC), the mean bias has been determined with respect to the theoretically expected value, see the main text.

**Table A4.**

*Mean estimation time (in minutes) per replication in each condition of the simulation study.*

| $\gamma$ | $N$ | $n$ |  | pJML | |  | cJML | |  | MCMC | |
| --- | --- | --- | --- | --- | --- | --- | --- | --- | --- | --- | --- |
|  |  |  |  | *Mean* | *SD* |  | *Mean* | *SD* |  | *Mean* | *SD* |
| 1 | 1,000 | 24 |  | 0.22 | 0.10 |  | 0.99 | 0.33 |  | 6.99 | 0.10 |
|  |  | 96 |  | 1.62 | 0.14 |  | 6.04 | 1.03 |  | 27.86 | 0.11 |
|  | 10,000 | 24 |  | 8.35 | 2.20 |  | 19.34 | 4.93 |  | 134.78 | 9.16 |
|  |  | 96 |  | 43.50 | 3.61 |  | 101.10 | 12.80 |  | 318.27 | 8.28 |
| 1.7 | 1,000 | 24 |  | 0.12 | 0.01 |  | 0.92 | 0.14 |  | 7.09 | 0.02 |
|  |  | 96 |  | 1.16 | 0.07 |  | 5.25 | 0.37 |  | 28.09 | 0.14 |
|  | 10,000 | 24 |  | 5.83 | 0.95 |  | 15.54 | 2.28 |  | 140.42 | 7.71 |
|  |  | 96 |  | 17.26 | 1.96 |  | 66.65 | 6.07 |  | 327.50 | 8.32 |

*Note*. These means are given just as an indication, see the text

**Appendix B:**

**Simulation Study on the Cross-Validation Procedure**

***Design***

In this simulation study, we focus on the performance of our proposed model selection approach. To this end, similarly to the main Simulation Study reported in the manuscript, we simulate data according to the LSIRM in Equation 1 with $\gamma$ either $1$ or $1.7$ (as in Study 1) and with either $R=0$ (a Rasch model with local independence), $R=1$, $R=2$, and $R=3$. In addition we use either $N=1,000$ or $N=5,000$, and $n=24$ or $n=48$. True item and person parameter values are the same as in Study 1. We use $50$ replications for each condition. For each replication in each condition, we determine the $UCE, URE,$and $RSS$ using either $5$ or $10$ folds in the cross-validation and pJML estimation of the parameters.

**Table B1**

*True positive rates of the* $UCE$*,* $URE$*, and* $RSS$ *metrics used in a cross-validation with* $5$ *or* $10$ *folds (*$K)$*, for the conditions differing in the number of persons (*$N)$*, the number of items* $(n)$*, the true value of* $\gamma$*, and the dimensions of* $\boldsymbol{z}_{p}$ *and* $\boldsymbol{w}_{i}$ *(*$R$*).*

| $\gamma$ | $N$ | $n$ | $R$ |  | Number of Folds ($K$) | | | | | | |
| --- | --- | --- | --- | --- | --- | --- | --- | --- | --- | --- | --- |
|  |  |  |  |  |  | 5 |  |  |  | 10 |  |
|  |  |  |  |  | *UCE* | *URE* | *RSS* |  | *UCE* | *URE* | *RSS* |
| $1.0$ | $1,000$ | $24$ | 0 |  | 0.98 | 1.00 | 1.00 |  | 0.98 | 1.00 | 1.00 |
|  |  |  | 1 |  | 0.66 | 0.78 | 0.78 |  | 0.78 | 0.92 | 0.96 |
|  |  |  | 2 |  | 0.86 | 0.90 | 0.90 |  | 0.92 | 0.96 | 0.96 |
|  |  |  | 3 |  | 0.88 | 1.00 | 1.00 |  | 0.96 | 1.00 | 1.00 |
|  |  | $48$ | 0 |  | 1.00 | 1.00 | 1.00 |  | 0.98 | 1.00 | 1.00 |
|  |  |  | 1 |  | 0.96 | 1.00 | 1.00 |  | 0.96 | 1.00 | 1.00 |
|  |  |  | 2 |  | 0.98 | 1.00 | 1.00 |  | 1.00 | 1.00 | 1.00 |
|  |  |  | 3 |  | 1.00 | 1.00 | 1.00 |  | 1.00 | 1.00 | 1.00 |
|  | $5,000$ | $24$ | 0 |  | 1.00 | 1.00 | 1.00 |  | 1.00 | 1.00 | 1.00 |
|  |  |  | 1 |  | 0.92 | 0.94 | 0.94 |  | 0.98 | 0.94 | 0.94 |
|  |  |  | 2 |  | 1.00 | 1.00 | 1.00 |  | 0.98 | 1.00 | 1.00 |
|  |  |  | 3 |  | 1.00 | 1.00 | 1.00 |  | 1.00 | 1.00 | 1.00 |
|  |  | $48$ | 0 |  | 1.00 | 1.00 | 1.00 |  | 1.00 | 1.00 | 1.00 |
|  |  |  | 1 |  | 1.00 | 1.00 | 1.00 |  | 1.00 | 1.00 | 1.00 |
|  |  |  | 2 |  | 1.00 | 1.00 | 1.00 |  | 1.00 | 1.00 | 1.00 |
|  |  |  | 3 |  | 1.00 | 1.00 | 1.00 |  | 1.00 | 1.00 | 1.00 |
| $1.7$ | $1,000$ | $24$ | 0 |  | 0.98 | 1.00 | 1.00 |  | 0.92 | 1.00 | 1.00 |
|  |  |  | 1 |  | 0.86 | 0.98 | 0.98 |  | 0.94 | 1.00 | 1.00 |
|  |  |  | 2 |  | 1.00 | 1.00 | 1.00 |  | 0.90 | 1.00 | 1.00 |
|  |  |  | 3 |  | 1.00 | 1.00 | 1.00 |  | 1.00 | 1.00 | 1.00 |
|  |  | $48$ | 0 |  | 1.00 | 1.00 | 1.00 |  | 0.98 | 1.00 | 1.00 |
|  |  |  | 1 |  | 0.98 | 1.00 | 1.00 |  | 1.00 | 1.00 | 1.00 |
|  |  |  | 2 |  | 1.00 | 1.00 | 1.00 |  | 0.98 | 1.00 | 1.00 |
|  |  |  | 3 |  | 1.00 | 1.00 | 1.00 |  | 1.00 | 1.00 | 1.00 |
|  | $5,000$ | $24$ | 0 |  | 1.00 | 1.00 | 1.00 |  | 1.00 | 1.00 | 1.00 |
|  |  |  | 1 |  | 0.98 | 1.00 | 1.00 |  | 1.00 | 1.00 | 1.00 |
|  |  |  | 2 |  | 1.00 | 1.00 | 1.00 |  | 0.96 | 1.00 | 1.00 |
|  |  |  | 3 |  | 1.00 | 1.00 | 1.00 |  | 1.00 | 1.00 | 1.00 |
|  |  | $48$ | 0 |  | 1.00 | 1.00 | 1.00 |  | 1.00 | 1.00 | 1.00 |
|  |  |  | 1 |  | 1.00 | 1.00 | 1.00 |  | 1.00 | 1.00 | 1.00 |
|  |  |  | 2 |  | 1.00 | 1.00 | 1.00 |  | 1.00 | 1.00 | 1.00 |
|  |  |  | 3 |  | 1.00 | 1.00 | 1.00 |  | 1.00 | 1.00 | 1.00 |

*Note.* $UCE$: Unnormalized Classification Error; $URE$: Unnormalized ROC Error; $RSS$: Residual Sum of Squares

***Results***

True positive rates across the conditions with $\gamma=1$ are in Table B1 for the different metrics. Generally, for results indicate that the metrics considered can already distinguish well between the models for $\gamma=1$ irrespective of the number of items, number of persons, or number of folds considered. For the smaller data scenario ($N=1,000$, $n=24)$, the $UCE$ has difficulties correctly identifying the $R=1$ model for $5$ folds (true positive rate of 0$.66$). However using $10$ folds, the true positive rate increases to 0.78 which is more acceptable. For $\gamma=1.7$ all true positives were at least 0.98, with one exception of a true positive of 0.86 for the $UCE$ in case of $R=1$ and $K=5$.

As these results are not displayed above, it should be noted that our cross-validation procedure tends to overselect, and not underselect. That is, although generally, the correct model is picked most of the times, if an incorrect model is being selected, it is always a model with a larger $R$ than the true model. This is a known property of cross-validation in general (see Li et al., 2020), but it should be kept in mind when using cross-validation for dimensionality selection.

***JIC***

After a suggestion by an anonymous reviewer, we reanalyzed our simulated data from the design above and added the Joint-likelihood-based Information Criterium (JIC: Chen &Li 2022). The index is given by

$JIC = -2 \mathcal{l}\left( \hat{\boldsymbol{\theta}}\mathbf{,}\hat{\boldsymbol{\beta}}\mathbf{,}\hat{\mathbf{W}}\mathbf{,}\hat{\mathbf{Z}}\mathbf{,} | \mathbf{X} \right)+R\times max \left( N,n \right)\times log\left( \frac{Nn-m}{\max\left( N,n \right)} \right)$

where $\hat{\boldsymbol{\theta}}\mathbf{,}\hat{\boldsymbol{\beta}}\mathbf{,}\hat{\mathbf{W}}\mathbf{,}$ and $\hat{\mathbf{Z}}$ denote the JML estimates and $m$ denotes the number of missing data entries in $\mathbf{X}$**.** Everything else is as defined in the manuscript. Results (not displayed) indicated that the JIC performed poorly, even in the case of $n=48$. However, as the study by Chen and Li considered scenarios with a much larger $n/N$ ratio, we added a small separate condition with $\gamma=1.7$, $N=1000$ and $n=200$ which closer follows the scenario considered by Chen and Li. The results are in Table B2 below and show that in this setting, the JIC perfoms well in selecting the true model for both pJML and cJML.

**Table B2**

True dimensions of the latent space (R) and acceptance rates by the JIC for r=0,1,2, and 3 dimensions.

| $R$ | $r=0$ | $r=1$ | $r=2$ | $r=3$ |
| --- | --- | --- | --- | --- |
| *Penalized JML* | | | | |
| 0 | **1** | 0 | 0 | 0 |
| 1 | 0 | **0.8** | 0.2 | 0 |
| 2 | 0 | 0 | **1** | 0 |
| 3 | 0 | 0 | 0.02 | **0.98** |
| *Constrained JML* | | | | |
| 0 | **1** | 0 | 0 | 0 |
| 1 | 0 | **0.76** | 0.24 | 0 |
| 2 | 0 | 0 | **1** | 0 |
| 3 | 0 | 0 | 0 | **1** |

*Note*. Acceptance rates of the true model are in bold

***Conclusion***

Generally, the different models can be well separated by all three metrics. As for smaller number of items and subjects, a $K=1$ model may be false reject using 5 folds, one can best rely on 10 folds in such situations. If the number of items is large enough, the JIC can be considered as an alternative index, but, with caution as its derivation by Chen and Li (2022) assumes a asymptotic properties that may not hold for the present models.

**Appendix C**

**Additional results illustrations**

**Table C1**

| **item** | $\boldsymbol{\beta}_{\boldsymbol{i}\boldsymbol{1}}$ | | $\boldsymbol{\beta}_{\boldsymbol{i}\boldsymbol{2}}$ | | $\boldsymbol{\beta}_{\boldsymbol{i}\boldsymbol{3}}$ | | $\boldsymbol{\beta}_{\boldsymbol{i}\boldsymbol{4}}$ | |  | $\boldsymbol{w}_{\boldsymbol{i}\boldsymbol{1}}$ | | $\boldsymbol{w}_{\boldsymbol{i}\boldsymbol{2}}$ | |
| --- | --- | --- | --- | --- | --- | --- | --- | --- | --- | --- | --- | --- | --- |
|  | *Est.* | *SE* | *Est.* | *SE* | *Est.* | *SE* | *Est.* | *SE* |  | *Est.* | *SE* | *Est.* | *SE* |
|  | COMMUNALITY SCALE | | | | | | | | | | | | |
| reliable | 4.72 | 0.29 | 2.98 | 0.18 | -0.05 | 0.16 | - | - |  | 0 | - | 1.26 | 0.13 |
| honest | 5.57 | 0.41 | 4.65 | 0.29 | 2.52 | 0.16 | -0.29 | 0.15 |  | -0.37 | 0.10 | 1.06 | 0.13 |
| unscrupulous* | 5.39 | 0.39 | 4.24 | 0.26 | 2.75 | 0.18 | 1.19 | 0.16 |  | -0.99 | 0.15 | -0.55 | 0.13 |
| deceitful* | 4.56 | 0.30 | 3.10 | 0.18 | 1.73 | 0.13 | -0.06 | 0.12 |  | 0.08 | 0.08 | -0.75 | 0.09 |
| unintelligent* | 5.71 | 0.43 | 5.01 | 0.33 | 3.06 | 0.19 | 1.00 | 0.16 |  | -1.22 | 0.16 | -0.13 | 0.16 |
| obnoxious* | 5.47 | 0.42 | 4.67 | 0.31 | 2.65 | 0.16 | 0.27 | 0.12 |  | 0.65 | 0.11 | -0.36 | 0.10 |
| thankless* | 5.66 | 0.43 | 4.38 | 0.27 | 2.71 | 0.18 | 0.71 | 0.15 |  | 0.94 | 0.13 | -0.39 | 0.11 |
| unfriendly* | 5.56 | 0.43 | 4.69 | 0.31 | 3.14 | 0.19 | 0.44 | 0.14 |  | 0.77 | 0.13 | -0.42 | 0.10 |
| dependable | 5.73 | 0.43 | 5.02 | 0.33 | 3.42 | 0.20 | 0.37 | 0.15 |  | 0.13 | 0.12 | 1.13 | 0.14 |
| cruel* | 5.31 | 0.38 | 4.04 | 0.24 | 3.07 | 0.19 | 1.83 | 0.16 |  | 0.26 | 0.14 | -1.05 | 0.13 |
|  | DOMINANCE SCALE | | | | | | | | | | | | |
| apathetic* | 5.59 | 0.41 | 4.41 | 0.25 | 2.78 | 0.15 | 1.33 | 0.11 |  | 0 | - | -0.59 | 0.09 |
| timid* | 5.59 | 0.35 | 3.75 | 0.20 | 1.94 | 0.14 | -0.32 | 0.14 |  | 0.24 | 0.11 | 0.91 | 0.12 |
| dominant | 3.55 | 0.21 | 2.35 | 0.19 | 0.60 | 0.19 | -1.88 | 0.26 |  | 1.82 | 0.17 | 0.53 | 0.12 |
| dreamy* | 3.95 | 0.23 | 2.09 | 0.21 | 0.69 | 0.21 | -1.46 | 0.27 |  | -0.88 | 0.17 | -1.84 | 0.19 |
| inhibited* | 5.39 | 0.31 | 3.17 | 0.18 | 1.60 | 0.16 | -1.29 | 0.18 |  | -0.90 | 0.13 | 0.91 | 0.14 |
| reserved* | 5.54 | 0.32 | 3.41 | 0.20 | 1.48 | 0.17 | -0.93 | 0.19 |  | -1.16 | 0.15 | 0.78 | 0.12 |
| enterprising | 5.41 | 0.35 | 3.25 | 0.17 | 1.03 | 0.13 | -1.83 | 0.19 |  | 1.00 | 0.11 | -0.23 | 0.10 |
| strong | 5.71 | 0.35 | 4.01 | 0.24 | 1.34 | 0.20 | -1.30 | 0.24 |  | 1.73 | 0.20 | -0.36 | 0.16 |
| withdrawn* | 5.19 | 0.30 | 3.15 | 0.18 | 1.38 | 0.15 | -0.72 | 0.15 |  | -0.60 | 0.10 | 0.92 | 0.13 |
| shy* | 4.93 | 0.27 | 2.84 | 0.17 | 1.12 | 0.15 | -1.05 | 0.17 |  | -0.13 | 0.12 | 1.26 | 0.13 |

*Parameter estimates and standard errors for the item parameters in the garded response LSIRM fit to the ACL data*

*Note.* Items with an asterisk in the name are contraindicative items for the corresponding scale and are reversely coded in the analyses. In addition, for “reliable” the lowest response catergory was not used, as a result, this item only has three threshold parameters.
